# OptiFlex: Multi-Frame Animal Pose Estimation Combining Deep Learning With Optical Flow

**DOI:** 10.3389/fncel.2021.621252

**Published:** 2021-05-28

**Authors:** XiaoLe Liu, Si-yang Yu, Nico A. Flierman, Sebastián Loyola, Maarten Kamermans, Tycho M. Hoogland, Chris I. De Zeeuw

**Affiliations:** ^1^Faculty of Mathematics, University of Waterloo, Waterloo, ON, Canada; ^2^Department of Neuroscience, Erasmus MC, Rotterdam, Netherlands; ^3^Netherlands Institute for Neuroscience, Royal Academy of Arts and Sciences, Amsterdam, Netherlands; ^4^Department of Biomedical Physics and Biomedical Photonics, Amsterdam UMC location AMC, University of Amsterdam, Amsterdam, Netherlands

**Keywords:** behaviour analysis, video analysis, motion tracking method, markerless tracking, deep learning, optical flow

## Abstract

Animal pose estimation tools based on deep learning have greatly improved animal behaviour quantification. These tools perform pose estimation on individual video frames, but do not account for variability of animal body shape in their prediction and evaluation. Here, we introduce a novel multi-frame animal pose estimation framework, referred to as OptiFlex. This framework integrates a flexible base model (i.e., FlexibleBaseline), which accounts for variability in animal body shape, with an OpticalFlow model that incorporates temporal context from nearby video frames. Pose estimation can be optimised using multi-view information to leverage all four dimensions (3D space and time). We evaluate FlexibleBaseline using datasets of four different lab animal species (mouse, fruit fly, zebrafish, and monkey) and introduce an intuitive evaluation metric—adjusted percentage of correct key points (aPCK). Our analyses show that OptiFlex provides prediction accuracy that outperforms current deep learning based tools, highlighting its potential for studying a wide range of behaviours across different animal species.

## Introduction

To make meaningful inferences about how the brain controls behaviour, precise quantification is essential. Whereas, humans can make qualitative inferences on animal behaviour, computational approaches are required for precise quantification of body positions and movements. A major challenge is to reliably extract quantitative descriptions of behaviour that can be used for downstream tasks, such as motion clustering (Wiltschko et al., 2015; Batty et al., 2019), sensorimotor correlations (Hoogland et al., 2015; Katsov et al., 2017; Streng et al., 2018; Mu et al., 2019) or 3D shape reconstruction (Biggs et al., 2018). This problem can be addressed by articulated animal pose estimation, which consistently tracks predetermined key points on a given animal. The key points can be any identifiable feature on the animal body, usually joints.

Although key point tracking can be done with great accuracy through human labelling on a frame-by-frame basis, it usually incurs a considerable time and labour cost, limiting the size of annotated datasets. Consequently, the need for accurate, fast, and scalable animal pose estimation has driven several efforts to automate animal pose estimation using both marker-based and markerless tracking. Marker-based tracking of key points usually involves placing reflective markers that can be detected with a camera system (Leblond et al., 2003; Moriuchi et al., 2019). Alternatively, one can use accelerometers to directly readout movement acceleration (Venkatraman et al., 2010; Silasi et al., 2013; Pasquet et al., 2016). Marker-based tracking has the advantage of providing straightforward processing of object location and animal pose (Mimica et al., 2018). However, its invasive nature could disrupt animal behaviour.

Markerless tracking circumvents the stress and workload associated with marker placement and could be the method of choice, if it matches the accuracy of marker-based tracking. Early examples of markerless tracking used Kinect cameras (Matsumoto et al., 2013) or multi-camera systems (Matsumoto et al., 2013), setting constraints on the simplicity and versatility of the experimental setting in which animal behaviour could be measured. Advances in deep learning based computer vision techniques, especially convolutional neural networks, have enabled further development of markerless tracking (Kanazawa et al., 2019; Kocabas et al., 2019; Pavllo et al., 2019; Sturman et al., 2020). For example, DeepLabCut (Mathis et al., 2018) is based on the feature detector from DeeperCut (Insafutdinov et al., 2016), and StackedDenseNet from DeepPoseKit (Graving et al., 2019) is a variation on Fully Convolutional DenseNets (Huang et al., 2017; Jégou et al., 2017) that are stacked in the fashion of Stacked Hourglass (Newell et al., 2016).

The general functioning of these deep learning based markerless pose estimation methods for animals are similar. First, they all require the user to manually label a number of key points on selected frames of animal behaviour videos. These video frames and location labels form the dataset used to train deep learning models. The dataset is divided into three groups, for training, validation and testing. For model training, a video frame is fed into the model as input, and the model output will be the model's prediction of key point locations. The difference between model predictions and the user generated labels are measured using a differentiable loss function, commonly mean squared error (MSE). After each iteration of input and comparisons, the model updates its parameters using the backpropagation algorithm, attempting to minimise future loss. The training is complete once the loss function value reaches a satisfactory level. After training, the model's prediction performance is measured using an evaluation metric, which is a function that compares model predictions against human labels and summarises model prediction accuracy with numerical values.

These approaches have brought meaningful advances to animal pose estimation (Sturman et al., 2020) by directly transferring computer vision techniques initially developed for tracking humans to lab animals, but omit key differences in shape and size. For pose estimation, the number of key points has a major impact on the size and complexity of pose estimation models and variation in key point sizes affects the interpretation of evaluation results.

Specifically, key points on humans are usually joints of limbs and the head, which are all roughly 20–50 cm in diameter (within 2x ratio). Since body shape does not vary drastically across humans, human pose estimation datasets usually contain limited variations in number or size of key points. Key points on lab animals, on the other hand, usually vary greatly in size. For instance, for a fruit fly, the joints on its legs are roughly 0.1–0.5 mm, while the key point on its head is roughly 2–3 mm (can be above 10x ratio). As a consequence, human pose estimation models capture similar amounts of complexity and evaluation metrics can use the size of a specific joint as a threshold. For instance, one common metric in human pose estimation is that a key point estimation is correct if its distance from the ground truth is less than half the size of the head (PCKh@0.5) (Newell et al., 2016; Chen et al., 2017; Yang et al., 2017). Lab animals, however, vary greatly in shape and size, requiring models with different complexities and parameter sizes to match varying animal datasets to prevent overfitting and underfitting. Moreover, since training datasets for animal pose estimation often need to be generated by researchers upon completion of experiments, these caveats are much more likely to emerge in animal research than in human studies, which usually can leverage plenty of annotated data sets. For example, COCO human key point detection task has more than 200,000 labelled images (Lin et al., 2014, 2018).

**Figure 1:**
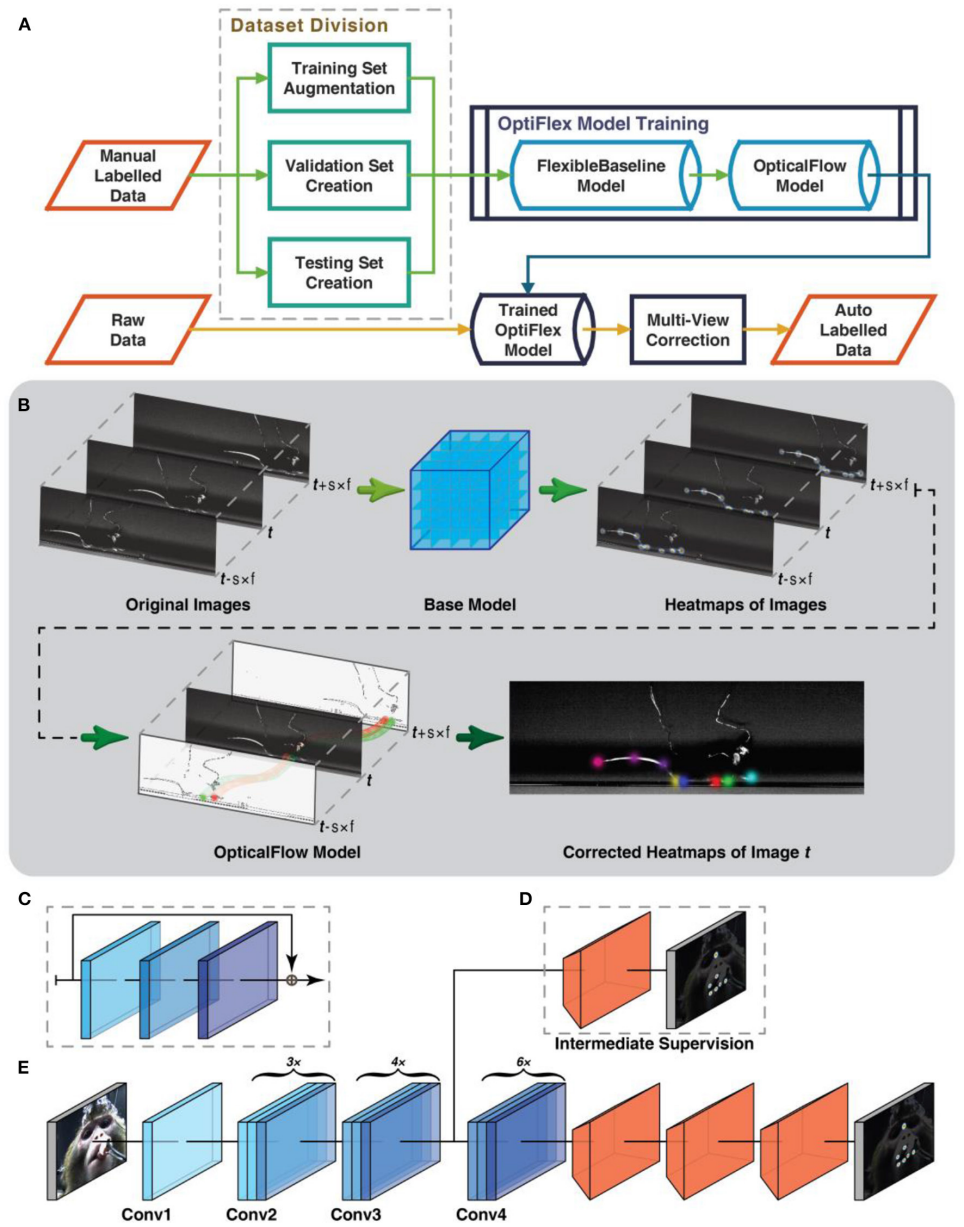
Workflow and model architecture of OptiFlex. **(A)** Overall data preprocessing and model training pipeline. **(B)** Given a skip ratio *s* and frame range *f*. For a target frame with index *t*, we first gather a sequence of 2*f* + 1 images with index from *t* − *s* × *f* to *t* + *s* × *f*. The base model makes a prediction on each of the images to create a sequence of heatmap tensors with index from *t* − *s* × *f* to *t* + *s* × *f*. The OpticalFlow model takes the entire sequence of heatmap tensors and outputs the final heatmap prediction for index *t*. **(C)** Diagram of a “bottleneck” building block commonly used in ResNet backbone, consisting of 3 convolutional layers and a skip connexion. **(D)** Optional intermediate supervision for FlexibleBaseline through an additional loss calculation between the heatmap label and intermediate results from ResNet backbone after a single transposed convolution as represented by orange trapezoidal blocks. **(E)** Standard FlexibleBaseline with intermediate supervision after the Conv3 block. Note that each Conv block consists of multiple (3x, 4x, or 6x) stacked “bottleneck” building blocks (see **C**).

To address the aforementioned differences in subject shape and data availability between animal and human pose estimation, we present OptiFlex, a new multi-frame animal pose estimation framework. OptiFlex is comprised of FlexibleBaseline and OpticalFlow. The base model, FlexibleBaseline, makes initial single frame predictions. The OpticalFlow model converges initial predictions on a target frame and its adjacent frames into a final prediction for the target frame (Figure 1B).

The base model, FlexibleBaseline, is designed to be flexible to accommodate various animal datasets that could include animals with different body shapes or varying number of key points. Compared to previous animal pose estimation models that have fixed model structure, FlexibleBaseline allows its model structure to be adjusted by changing its backbone output layer or the number of filters in its final deconvolution layers.

Most animal pose estimation frameworks to date (Mathis et al., 2018; Graving et al., 2019; Pereira et al., 2019) focus on predicting key points using a single video frame. By taking a single frame approach, these models neglect the sequential nature of these frames, and thus ignore valuable temporal context. Since the OptiFlex framework permits the concurrent use of features from adjacent time points in a video segment, both prediction accuracy and robustness against temporary obstruction of key points are improved. Moreover, the framework consists of two modules to allow swift deployment of the base model of choice. Rapid advancements in computer vision will result in better base models for single frame pose estimation in the near future, yet they can all potentially benefit from the temporal context provided by optical flow (Pfister et al., 2015).

Finally, to evaluate these models, we use datasets of four different lab animal species (mouse, fruit fly, monkey, and zebrafish) and an adaptive, yet intuitive, evaluation metric—adjusted percentage of correct key points (aPCK). Compared to the traditionally used root mean square error (RMSE) metric, aPCK encapsulates human insight of what is “correct” for each key point by leveraging human generated heatmap labels. In the end, aPCK reports a percentage of prediction in the “correct” region, which is more interpretable than the raw pixel value of average error distances reported by RMSE. All prediction performance evaluations in this study are done using aPCK.

## Results

Our overall workflow consists of a multi-frame estimation framework along with a graphical user interface (GUI) for data annotation and augmentation (Figure 1A, for details refer to Supplementary Figures 2,3).

### General Workflow

The workflow starts after obtaining video recordings of animal behaviours (Figure 1A). A human annotator first labels a number of continuous frames, which are then preprocessed (details in Data Preprocessing section of Methods) and subsequently split into three datasets: a train set, validation set, and test set. Models presented in this work are trained with the train set and evaluated on the validation set. These evaluation results are used not only to tune the hyperparameters of the models, such as learning rate, dropout rate, number of filters in convolution layers etc., but also to determine whether more labelled data should be obtained. The model with the best performance on the validation set is chosen as the final model. For OptiFlex, this training process happens twice, once for the base model, FlexibleBaseline, and once for the OpticalFlow model. After obtaining satisfactory evaluation results on the validation set, a final evaluation can be run on the test set to quantify how the trained model generalises to new data. All of the test datasets include frames of different animals of the same type to ensure generalizability of the prediction results. This marks the end of the training process. Any new videos of animals in the same experimental configuration can now be used as inputs into the trained model and the outputs will be the videos labelled with locations of user defined key points.

### FlexibleBaseline Structure of OptiFlex

FlexibleBaseline's overall structure consists of a ResNet (He et al., 2016) backbone, 3 transposed convolution layers, and a final output layer (Figures 1C–E). The ResNet backbone is a section of the original ResNet that uses weights pre-trained on ImageNet (Deng et al., 2009) and can output after any of the Conv blocks from ResNet. It also allows optional intermediate supervision anywhere between the input layer and the backbone output layer, usually after a Conv block. The different options for backbone output and intermediate supervision endow the model with ample flexibility. The 3 transposed convolution layers all have filters of size 13 × 13 pixels (i.e., the window for scanning through the input image or intermediate tensors is 13 × 13) with strides depending on output location of the ResNet backbone and with a modifiable number of filters in each layer depending on dataset complexity. The final output layer always has the same number of filters as the number of prediction key points, each with filter size 1 × 1 and stride 1. This structure is inspired by the Simple Baselines (Xiao et al., 2018), which attained state-of-the-art results in many human pose estimation challenges in COCO 2018 (Lin et al., 2014, 2018).

In principle, any imaged-based model that predicts a set of heatmaps of key point likelihoods can be used as a base model. Different options of FlexibleBaseline allow for adaptation to various animal body shapes. For animals with complex body shape, FlexibleBaseline versions with a large number of parameters can be deployed to learn the various features. For animals with simple body shape, FlexibleBaseline versions with fewer parameters are more suited, as smaller models usually have faster inference speed. This ability to change model complexity based on dataset complexity makes FlexibleBaseline less likely to overfit during training, and thus better at accounting for body shape variations in animal datasets.

### OpticalFlow Structure of OptiFlex

With single frame heatmap predictions from a base model, the OpticalFlow module morphs heatmap predictions of neighbouring frames onto the target frame using the Lucas-Kanade method (Lucas and Kanade, 1981), implemented with the Farneback algorithm (Farnebäck, 2003) in OpenCV (Bradski, 2000). This morphed information is aggregated by a 3D convolution layer that essentially takes the weighted sum of all the morphed heatmaps (Figure 1B). This implies that even if some key points are not visible in the target frame, the morphed information from nearby frames still provides sufficient information about the most likely location of the key points for the target frame. The morphed heatmaps from nearby frames can thus be considered as temporal context.

### Adjusted Percentage of Correct Key Points (aPCK) vs. Root Mean Square Error (RMSE)

We indicate the adjusted percentage of correct key points by aPCK, which is an evaluation metric that measures the percentage of correctly predicted key points based on human generated heatmap labels. The heatmap generated for each key point follows a truncated 2D Gaussian distribution with the human labelled points defining the peak of the distribution. For the current study we define the prediction as “correct,” if the predicted key point location lands inside of the ground truth label heatmap. For details of label preprocessing and heatmap generation process, see Dataset Preprocessing in Methods.

Predefined notion of PCK (Yang and Ramanan, 2011) in computer vision usually accepts all points within a fixed distance of the human labelled ground truth points (Biggs et al., 2018). While generic PCK is a valid approach for human pose estimation, where all human key points are similar in size, it cannot be flexibly applied to datasets of animals with different body plans, where the large size variation across key points of the same animal makes it impractical to use a single fixed distance to determine acceptable regions for every key point. By contrast, aPCK relies on the human labeller to define the acceptable region per key point with its heatmap, and allows adjustments of its size during labelling. More importantly, the universal nature of aPCK, as it adequately accounts for variability, makes model evaluation results from datasets with different animal species comparable. We therefore use aPCK as the default metric for evaluations in this study.

RMSE is currently the default evaluation metric in recent work on animal pose estimation (Mathis et al., 2018; Graving et al., 2019; Pereira et al., 2019). While RMSE can be useful as the loss function, this metric does not intuitively reflect prediction quality and can sometimes be misleading.

**Figure 2:**
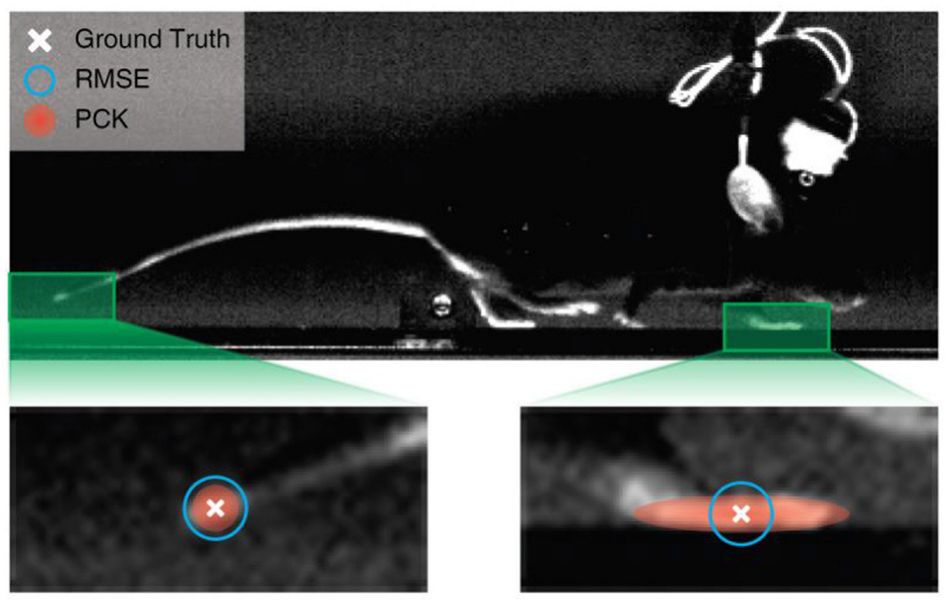
Comparison of evaluation methods. The ground truth is defined through human labelling. Points with a given RMSE forms a circle around the ground truth. The heatmap label, used by the adjusted percentage of correct key points (aPCK), can be defined by the human labeller to cover the entire ROI, whereas the RMSE circle can be too large and include points that fall outside the target region (in this case the paw) or too small and thereby miss valid points of the labelled object.

The preciseness of RMSE can be limited both when comparing the performance of different models using the same dataset and when comparing the performance of the same model applied to different datasets. When comparing two models using the same dataset, the model with a slightly larger RMSE is not necessarily worse. This is because some animal key points can be large (many pixels in size); therefore, predictions that differ by only a few pixels can both be correct. Moreover, since all points of a given RMSE from ground truth form a circle around the ground truth location, whereas an acceptable region for a key point can be of any shape, points with the same RMSE from ground truth can both be valid or incorrect predictions. For instance, in the example in Figure 2, some of the points within the blue circle representing RMSE threshold fall outside of the target region, and likewise correct points will be missed when the RMSE circle is shrunk. When comparing the same model using different datasets, RMSE fares even worse. A model with a given RMSE can make perfect predictions in a dataset with larger joint sizes, while being completely inaccurate in another dataset with smaller joint sizes. This combination of biases may explain why RMSE is often used as loss function, but not as evaluation method in human pose estimation (Newell et al., 2016; Chen et al., 2017; Yang et al., 2017).

### OptiFlex Compared to Previous Frameworks

**Figure 3:**
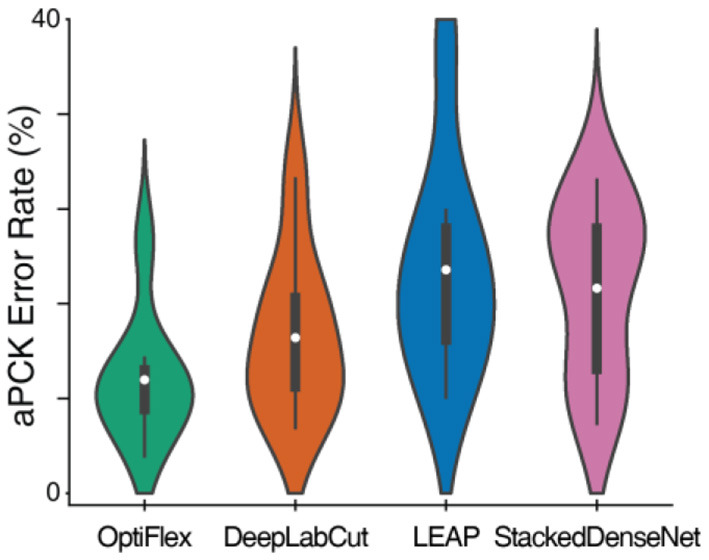
Comparison of aPCK error rates of various frameworks on the mouse side-view dataset displayed as a violin plot. The white dots represent the mean; the dark grey boxes represent the range between 1st quartile and 3rd quartile; and the whiskers represent the variability outside the upper and lower quartiles. The violin plots are aggregations of key point prediction error rates of a given model on the mouse side-view test set (1,051 frames from different videos with 8 labelled key points). Each data point represents the aPCK error rate of a particular model's prediction of a specific key point on the test set of mouse side-view dataset.

To demonstrate the potential of temporal context, we compare OptiFlex to current state-of-art animal pose estimation models: DeepLabCut (Mathis et al., 2018), LEAP (Pereira et al., 2019), and DeepPoseKit (StackedDenseNet) (Graving et al., 2019). The best dataset for highlighting the effect of temporal context is the mouse side-view dataset. This dataset is particularly interesting, because frequent overlap of paws causes many instances of temporary obstruction of key points, which is an issue that has plagued previous frameworks. Thus, we benchmarked the performance of various frameworks on the mouse side-view dataset (see Figure 3, Table 1). Our results show that OptiFlex significantly outperforms previous frameworks on every key point in a dataset with frequent instances of temporary obstructions, illustrating the importance of temporal context.

**Table 1:** Comparison of OptiFlex with previous frameworks on mouse side-view dataset.

**Joint Name**	**OptiFlex**	**DeepLabCut**	**LEAP**	**StackedDenseNet**
Front Right Paw	0.070409	0.086584	0.214082	0.264510
Hind Right Paw	0.099905	0.131304	0.169363	0.210276
Front Left Paw	0.114177	0.163654	0.238820	0.198858
Hind Left Paw	0.085633	0.172217	0.171265	0.206470
Snout	0.085633	0.089439	0.125595	0.103711
Tail 01	0.031399	0.055186	0.127498	0.058991
Tail 02	0.213130	0.265461	0.381541	0.242626
Tail 03	0.065652	0.087536	0.080875	0.099905
***BEST COUNT***	**8**	0	0	0
***MEAN***	**0.095742**	0.131423	0.188630	0.173168
***SD***	**0.053491**	0.067803	0.092872	0.075159
***Paired t-test p-value***	N/A	**0.00628**	**0.00143**	**0.00948**

*Best results at each joint are marked as GREEN, best statistical results are marked as GREEN_BOLD, all error rates are calculated using aPCK*.

*Bold values indicates to emphasize these values are showing the statistic significance*.

### Improving Robustness Against Temporary Obstruction With OpticalFlow

**Figure 4:**
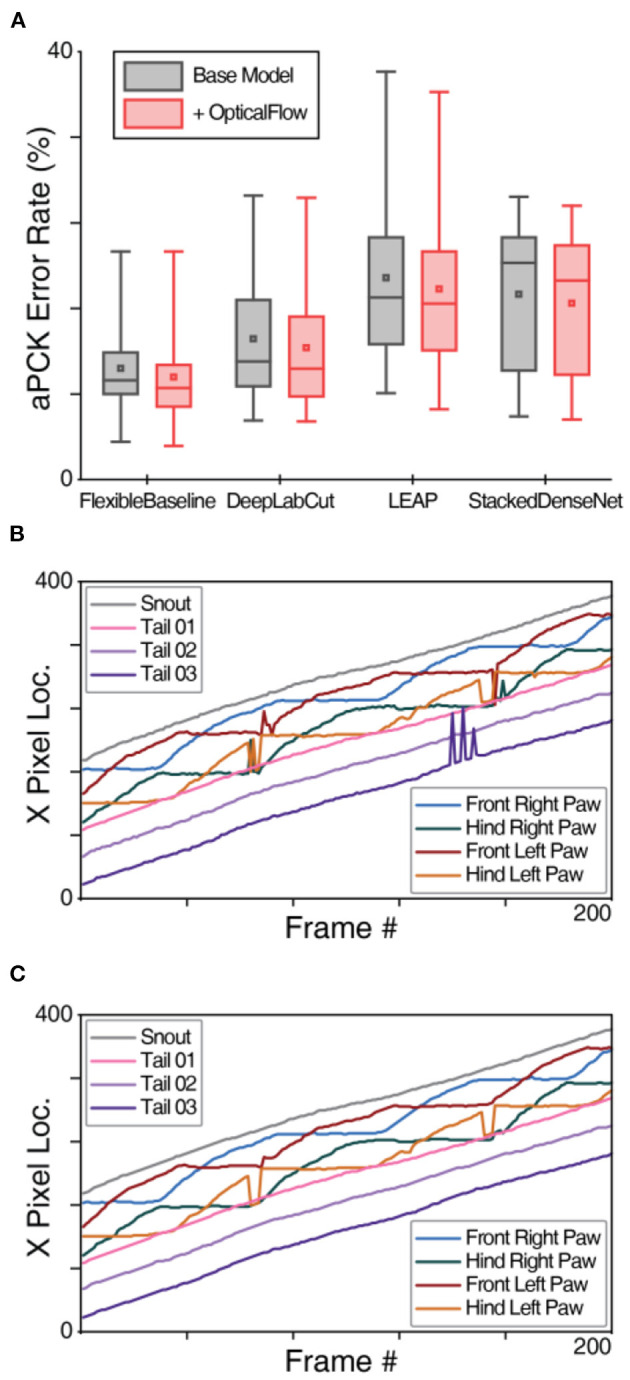
OpticalFlow model of OptiFlex evaluation. **(A)** Box plot of test set prediction aPCK error rates of models with and without OpticalFlow grouped by base model. Box plot specified with minimum, first quartile, median, third quartile and maximum; the small square in each box represents the mean. The benefits of the OpticalFlow model appeared universal in that it also improved the performance of the other base models (LEAP, DeepLabCut, and StackedDenseNet). The box plots are aggregations of key point prediction error rates of a given model on the mouse side-view test set (1,051 frames from different videos with 8 labelled key points). Each data point represents the aPCK error rate of a particular model's prediction of a specific key point on the test set of mouse side-view dataset. **(B)** X-value traces of FlexibleBaseline key point predictions for a mouse side-view video without OpticalFlow correction. **(C)** X-value traces of OpticalFlow model key point predictions for the same mouse side-view video (using FlexibleBaseline). Note that the differences after applying the OpticalFlow model are most prominently reflected in the smoothing of the trace curves of the key points. Sharp spikes in the trace curves correspond to prediction errors detected by OpticalFlow.

Our finding that OptiFlex benefits from the temporal context aggregated by the OpticalFlow model raises the question to what extent the performance of other base models can also be enhanced by applying the OpticalFlow model. To explore this, we added the OpticalFlow model also to DeepLabCut, LEAP, and StackedDenseNet. Even though integrating OpticalFlow model with FlexibleBaseline (i.e., OptiFlex) produced the best results, addition of the OpticalFlow model improved performance of all available base models (Figure 4A, Supplementary Table 5A, Supplementary Video 1). We compared prediction performances with and without OpticalFlow using paired *t*-tests and reported the significance of all comparisons in Supplementary Table 5B of the Supplementary Information. Results of OpticalFlow corrections are most evident through smoothing of trace curves of the keypoints. Sharp spikes in the trace curves were model prediction errors, and the OpticalFlow curve comparisons in Figures 4B,C provide good examples of where the spikes are smoothed out by OpticalFlow, indicating error correction. It should be noted that when the base model makes multiple consecutive erroneous predictions, the OpticalFlow model does not recognise those predictions as temporary obstructions, and does not make corrections (see e.g., Hind Left Paw in Figure 4C).

### Exploration in Multi-View Correction

Instead of using multiple views to construct 3D representation of joint movements, we explored the idea of using heatmap predictions obtained from different views to correct each other. Exploiting multiple views of the same behaviour can improve the predictions, because certain features are more identifiable in one view than another and the geometrical configuration of the different views determines the information shared between them. To demonstrate the potential of multi-view correction, we developed a simple algorithm that corrects paw predictions in the mouse dataset using initial predictions from both the side and bottom-views.

For the mouse dataset, the two perpendicular views (side and bottom) must share an axis, i.e., the x-axis, in 3D space. By comparing the single view prediction results (Supplementary Tables 7A,B), it could be determined that the bottom-view model better predicted the position of the paws. As a consequence, the x-value of the paws from the bottom could be used as a reference to search for alternative prediction locations for the paws in the side-view. These alternative prediction locations were generated by finding local maximums in the prediction heatmap using Gaussian filters in the side-view. Finally, the optimised locations for the paws in the side-view corresponded to the locations with the least difference in x-value from their respective key points in the reference (bottom) view.

**Figure 5:**
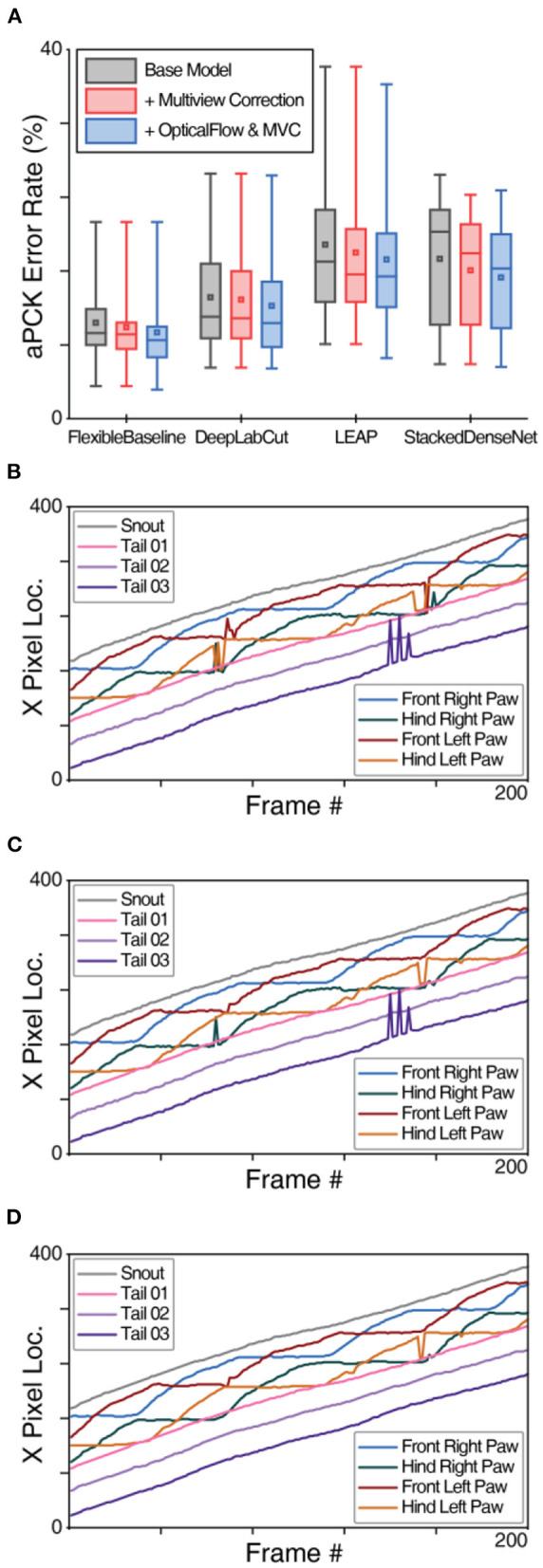
Multiview paw correction algorithm evaluation of OptiFlex. **(A)** Box plot of test set prediction aPCK error rates of models with and without multi-view correction on paws, grouped by base model. Box plot specified with minimum, first quartile, median, third quartile and maximum; the small square in each box represents the mean. Refer to Figure 4A caption for details of the data represented in the box plot. **(B)** X-value traces of FlexibleBaseline paw predictions for a mouse side-view video. **(C)** X-value traces of FlexibleBaseline paw predictions for a mouse side-view video after multi-view correction. **(D)** X-value traces of OptiFlex, which integrates FlexibleBaseline and OpticalFlow, paw predictions for the same mouse side-view video (video code: 00000nst_0028_tst) after multi-view correction.

Using the multi-view correction algorithm improves predictions for all base models. OptiFlex, integrating FlexibleBaseline and OpticalFlow, with multi-view corrections achieves the overall best results. We compared prediction performances with and without multi-view correction using paired *t*-tests and report the significance of all comparisons in Supplementary Table 6B. We also demonstrate the effects of multi-view correction in Figure 5, Supplementary Table 6A, Supplementary Video 2. Sharp changes in the trace curves in Figures 5B,C were model prediction errors, and most of these prediction errors were fixed after applying multi-view correction as shown in Figure 5D.

### Generalizability of FlexibleBaseline to Different Animals

**Figure 6:**
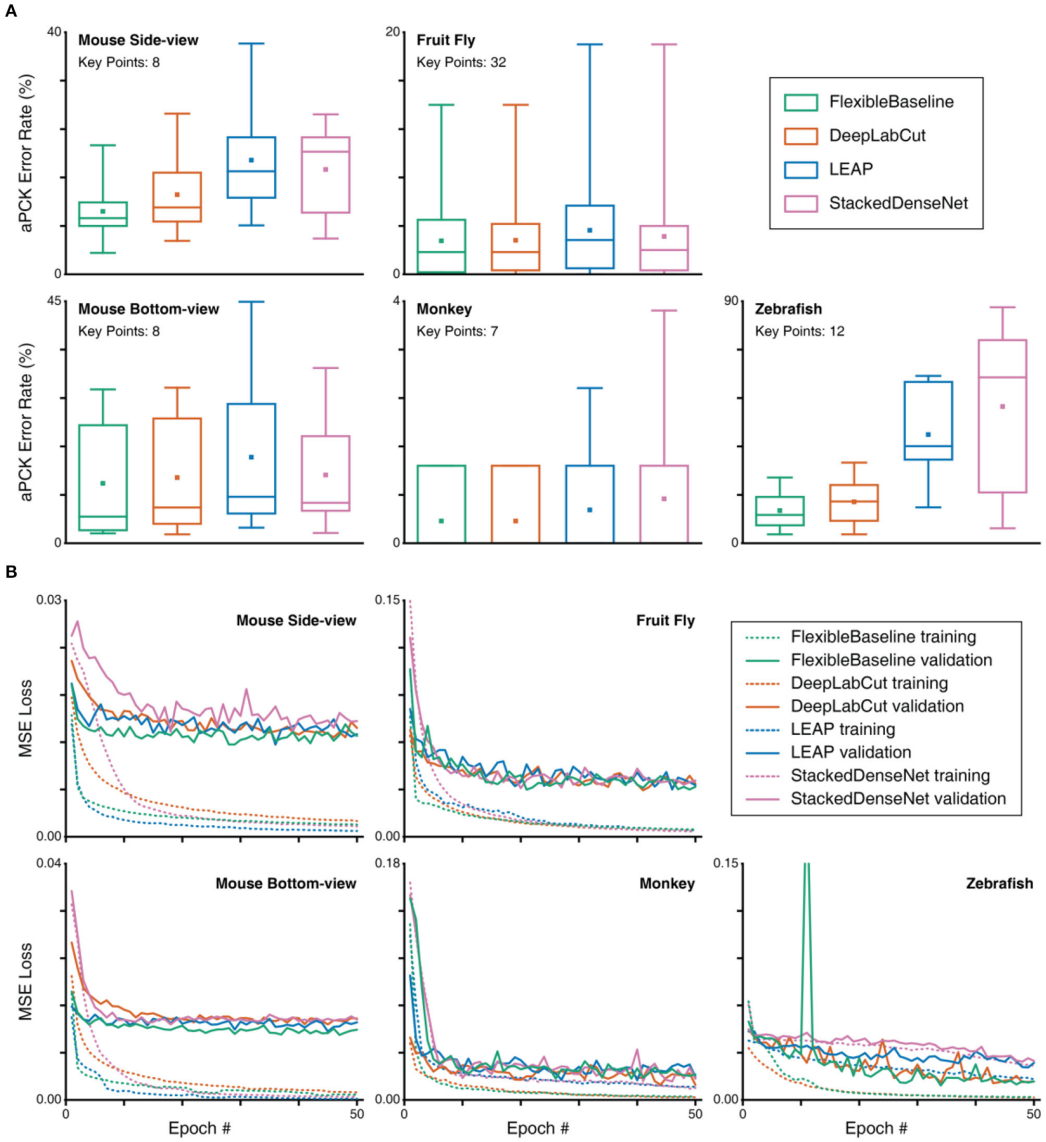
Comparison of aPCK error rates and MSE loss curves on different datasets. **(A)** Test set prediction error rates represented in box plots. Box plot specified with minimum, first quartile, median, third quartile, and maximum; the small square in each box represents the mean. Note that some monkey results do not show whiskers (parameters of variability), due to nearly perfect predictions. Refer to Figure 4A caption for details of the data represented in the box plot. The number of frames in the test set of each animal are specified in Supplementary Table 2, and the number of key points for each animal is indicated on the plot. **(B)** MSE training and validation loss; it should be noted that the peak in zebrafish reflects the fact that the optimizer landed in a poor spot early in the training process.

To demonstrate the generalizability of OptiFlex's base model, FlexibleBaseline, to different animals, we compared FlexibleBaseline to current state-of-the-art animal pose estimation models (DeepLabCut, LEAP, and DeepPoseKit) on datasets of four different animals. For all datasets, FlexibleBaseline achieved the best performance amongst all of the models in terms of lowest mean prediction error rate (Figure 6), with an error rate of only 0.0276 for the fruit fly dataset and 0.0370 for the monkey facial expression dataset. The largest difference in mean error rate compared to the next best model, DeepLabCut, was found for the mouse side-view and zebrafish dataset, with a 21.08% decrease (0.1213 vs. 0.1537) in error rate for the zebrafish dataset and a 20.93% decrease (0.1039 vs. 0.1314) in error rate for the mouse side-view dataset. In addition to overall prediction error rates, we also provide more granular statistics for detailed comparisons. These include the prediction accuracy rate for each of the key points, counts of the number of best predicted key points for each model (Supplementary Tables 7A–E), and paired *t*-test results between FlexibleBaseline and all other models to demonstrate the significance of our results (Supplementary Table 7F). Most notably, FlexibleBaseline also has the highest number of best predicted key points across all datasets. The most significant differences between FlexibleBaseline and DeepLabCut happens in the mouse side-view dataset (20.93% decrease in error rate, at *p*-value of 0.02663) and mouse bottom-view dataset (8.70% decrease in error rate, at *p*-value of 0.00622). More detailed comparisons of model accuracy are provided in Supplementary Information and a video comparison of tracking results is shown in Supplementary Videos 3A–D.

The inference speed of FlexibleBaseline was measured as the time the model takes to predict heatmaps from preprocessed input tensors of a particular dataset. The measurements were done on virtual machine instances (VM instances) of identical configuration on Google Cloud (see Computing Environment). To account for potential variability, the same prediction process was run 16 times, and the final results reflect the averages of these runtimes. For real-time inference with a batch size of 1, FlexibleBaseline had an average per image inference speed of 35 ms for the fruit fly test set, 18 ms for the monkey test set, 25 ms for the zebrafish test set, 12 ms for the mouse bottom-view test set, and 14 ms for the mouse side-view test set. For larger batch sizes, inference speed can still increase. For example, with a batch size of 128, FlexibleBaseline has an average per image inference speed of 26 ms for the fruit fly test set, and with a batch size of 256, FlexibleBaseline has an average per image inference speed of 16 ms for the monkey test set, 24 ms for the zebrafish test set, 6 ms for the mouse bottom-view test set, and 8 ms for the mouse side-view test set.

### Flexibility of FlexibleBaseline in Resource Constrained Situations

The flexibility of FlexibleBaseline derives from the fact that a user can select output from any of the 5 Conv blocks from ResNet50 and specify the number of filters in the last three transpose convolution layers. Different combinations of output block and filter numbers can vary greatly in the number of parameters, and thereby training and inference speed, while retaining a comparable accuracy across datasets. This flexibility gives users a very favourable trade-off between speed and accuracy when necessary.

**Figure 7:**
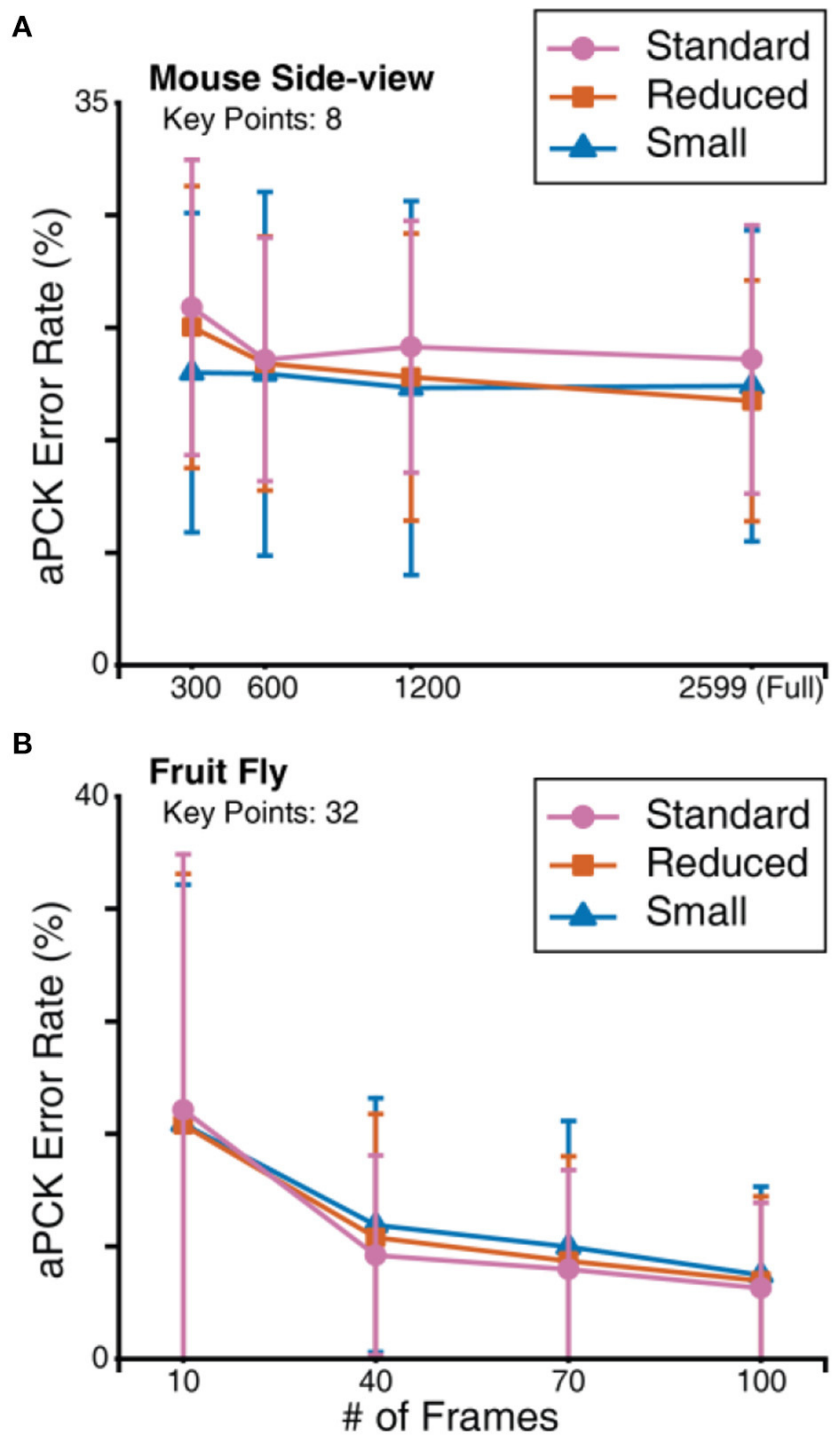
FlexibleBaseline model size evaluation. **(A)** Prediction aPCK error rate on sampled mouse side-view dataset. **(B)** Prediction aPCK error rate on sampled fruit fly dataset. To simulate hardware constraints, all mouse side-view models were trained for 40,000 steps at a batch size of 10, and all fruit fly models were trained for 8,000 steps at a batch size of 10.

Resource constrained situations are situations where not enough labelled data are available or the hardware does not support a large number of epochs. To simulate these conditions, we tested FlexibleBaseline with 3 different hyperparameter settings using a considerably reduced number of training steps and a minimal amount of annotation on the mouse side-view (Figure 7A) and fruit fly (Figure 7B) datasets. The number of parameters in these models decreased from more than 25 million in the standard version to <2 million in the small version (see Methods for detailed model and training setup, and Supplementary Table 9, Supplementary Figure 1 for performance of the 3 versions under the previous non-constrained training setup).

Recent animal pose estimation models (Graving et al., 2019; Pereira et al., 2019) suggest that reasonable accuracy can be achieved with as few as 100 labelled frames on the fruit fly dataset. We thus started with only hundreds of frames for both datasets. Independent from the versions of FlexibleBaseline, training with 100 frames in the fruit fly dataset already yielded fairly low prediction error rates: ~5% mean error rates or ~95% mean accuracy (Figure 7B, Supplementary Table 8B). When we gradually reduced the number of labelled frames, we observed a natural increase in prediction error rates with all versions. The standard version of FlexibleBaseline had the lowest error rate on the vast majority of the tested datasets. In case of the mouse side-view dataset (Figure 7A, Supplementary Table 8A), the models had higher prediction error rates with 300 labelled frames, so we explored training with a geometrically increased number of labelled frames. Our results indicated that under resource constrained training setups, small, and reduced versions outperformed the standard version in all of the tested mouse side-view datasets, while prediction error rates showed a plateau beyond 600 labelled frames.

## Discussion

This paper introduces a novel multi-frame estimation framework for animal pose estimation, which we refer to as OptiFlex. We exploit a new universal metric, adjusted percentage of correct key points or aPCK, to evaluate performance in the context of animal pose estimation. We show that our base model, FlexibleBaseline, achieves the lowest prediction error rates compared to other commonly used models for animal pose estimation when applied to behavioural datasets of four different animal species. Our FlexibleBaseline model can be further enhanced by leveraging the temporal context information through our OpticalFlow model (i.e., OptiFlex) to correct for temporarily obstructed key points. Moreover, we demonstrate that multi-view corrections can be added to further reduce key point prediction errors. Overall, the effectiveness and robustness of our OptiFlex framework, which is available through our open source Github repository (https://github.com/saptera/OptiFlex), make it a potentially valuable component in any system that wishes to track animal behaviour.

### Comparison of OptiFlex With Previous Deep Learning Based Animal Pose Estimation Frameworks

Compared with previous frameworks, OptiFlex has a more flexible base model, and considers more contextual information through the OpticalFlow model. Thus, the better performance of OptiFlex originates from the combination of FlexibleBaseline and OpticalFlow.

In terms of base models, FlexibleBaseline already achieved some improvements in mean error rate over other models (Supplementary Tables 7A–E). This improvement can be attributed to a better model architecture. DeepLabCut had comparable performance to FlexibleBaseline because they share the same ResNet backbone and both use a final deconvolution layer to generate heatmaps (He et al., 2016; Mathis et al., 2018). The main difference between DeepLabCut and FlexibleBaseline is that FlexibleBaseline does not always use the full ResNet backbone and has a few extra deconvolution layers at the end. Also, all other frameworks (DeepLabCut, LEAP, and DeepPoseKit) have one model setting for all animals (Mathis et al., 2018; Graving et al., 2019; Pereira et al., 2019), while FlexibleBaseline allows adjustments of model structure based on dataset complexity. For example, in complex datasets with large variation in key point features or with a large number of key points, FlexibleBaseline can be adjusted to include more pre-trained layers of the ResNet backbone and larger number of filters in the deconvolution layers. These adjustments will increase the overall number of trainable parameters in the model structure, allowing the model to better adapt to a larger variation in features or larger number of key points.

The other source of improvement is our OpticalFlow model. The accuracy improvement from this model can be attributed to the temporal context aggregated from the initial predictions of adjacent frames. With regards to temporal context, all other frameworks make predictions based on information from single frames, while OptiFlex incorporates temporal context from multiple frames by combining multiple FlexibleBaseline predictions with the OpticalFlow model. We also showed that applying OpticalFlow can be beneficial not only for FlexibleBaseline of OptiFlex, but also in combination with all other base models that we tested. In all cases, it was particularly instrumental in correcting for temporary obstruction. The issue of temporary obstruction has been identified before by others, and it has been partly circumvented by applying a variety of strategies (Graving et al., 2019; Nath et al., 2019; Pereira et al., 2019). For example, some models avoided temporary obstructions by analysing datasets that had a relatively high visibility of the key points (Graving et al., 2019; Nath et al., 2019), while others did this by cutting off the error distances at the 90th percentile level (Pereira et al., 2019).

### Dataset Considerations

The diverse body plans of different species and distinct experimental setups in the datasets used for assessment of our model posed unique challenges. The mouse dataset was obtained on a setup with both side and bottom-views, allowing the combination of spatial geometric information from both views. In the mouse side-view dataset, limb alternation during locomotion was associated with temporary obstruction of paws making continuously tracking of each paw a challenge. Thus, this dataset provided an ideal test case for our OpticalFlow approach in improving model predictions with temporary obstruction of key points.

The fruit fly dataset on the other hand, which was from the same dataset as used by LEAP (Pereira et al., 2019), had the largest number of key points to track, resulting in a higher memory load for models as compared to the other datasets. At the same time, the tracking process was simplified by removal of image backgrounds and high visibility of the key points. The monkey dataset comprised only facial features, with one of the key points, the tongue, rarely appearing in the dataset. This feature made the monkey dataset suitable for testing datasets with highly imbalanced key point occurrences. The zebrafish dataset, finally, was the only dataset that required tracking of multiple animals at the same time. This endeavour was particularly challenging as individual zebrafish are hard to distinguish, while they frequently traverse the field of view. At present, OptiFlex can only track multiple animals when the number of animals is predetermined and the body shape of each animal is consistent throughout the dataset. This ability could be generalised to track an arbitrary number of animals by adding an extra object detection module, which is a potential direction for future work. Taken together, the data of the different species comprised a rich and diverse set of behavioural measurements that allowed us to test the benefits and limitations of OptiFlex and other state-of-the art pose estimation models to their full extent.

### Generalisation of Multi-View Correction

Our exploration of multi-view correction only involves two views, but this approach can be generalised to recordings with more than one view for most setups. There are existing bootstrap methods in computer vision that use prior knowledge of camera setup and triangulation to aggregate positional information of a given key point from multiple views (Simon et al., 2017). More specifically, their approach first collects 2D location of each key point from noisy initial predictions of the key point in multiple views. Since the camera angles for each view is known, these 2D locations can be used to triangulate potential key point locations in 3D space. After using an algorithm to determine the mostly likely 3D location of each key point, the final 3D location can be reprojected back to each view as the corrected 2D location for the key point on each view. This way, even when key points are not clearly visible in some views, locations of those key points can be confirmed in other views where they are precisely identified through 3D triangulation and reprojection.

### Spatial, Geometrical, and Temporal Context

Fundamentally, animal key point movements happen in 3D space over time, making the intrinsic information four dimensional. If predictions are made on animal key point movements using only two-dimensional data, such as a single video frame, then 2 dimensions of available information are forfeited. This, fundamental flaw will persist despite improvements in models or training datasets. Here, we show that all 4 dimensions can be taken into account by using FlexibleBaseline to generate predictions based on 2D video frames, by combining multi-view analysis to generate predictions in a 3D geometric context, and by adding the OpticalFlow model to utilise the fourth temporal dimension. By integrating all these features into a single framework, OptiFlex provides the next step forward to use advanced deep learning tools to analyse animal behaviour non-invasively at a high spatiotemporal resolution.

## Methods

### Formulation

The goal of OptiFlex is to produce a set of heatmaps representing the model's confidence of each key point appearing at each location of the image. We denote the pixel location of the *p*^*th*^ key point as *Y_p_ ∈ Z* ⊂ ℝ^2^, where *Z* is the set of all (*x, y*) locations in an image and *p*∈{1 ... *P*}.

The base model has *N* outputs, each considered as a function. With the *i*^*th*^ output denoted by *b*_*i*_(·), where *i* ∈ {1 ... *N*}. Usually, there are 1 to 2 outputs obtained from the base model. Each output goes through a resizing process (usually deconvolution), denoted *d*_*i*_(·), to produce a set of intermediate heatmaps **h**_*i*_. Thus, if we let the input frame be denoted as **x**, we have $d_{i}\left(b_{i}\left(x\right)\right)\;\to {\left\{{\text{h}}_{i}^{p}\right\}}_{p\;\in \;\left\{1\;...\;P\right\}}={\text{h}}_{i}$, where ${\text{h}}_{i}^{p}$ is the heatmap for the *p*^*th*^ key point from the *i*^*th*^ output. The intermediate heatmaps **h**_*i*_ are used to compare against labels for intermediate supervision, and the final set of heatmaps **h**_*N*_, denoted **f**, is used as the output heatmap for the base model.

To use context information from surrounding frames, the base model was first applied to predict heatmaps for all surrounding frames. Let ${\left\{{\text{x}}^{k}\right\}}_{k\;\in \;\left\{t-s\times f\;...\;t\;...\;t+s\times f\right\}}$ be a sequence of input frames, where *t* is the index of the target frame, *s* is the skip ratio, and **f** is the frame range. We applied the base model to each frame to get the output heatmaps $d_{N}\left(g_{N}\left({\text{x}}^{k}\right)\right)={\text{f}}^{k}$. Next, we computed optical flow between each of the surrounding frames and target frame **x**^*t*^, then we applied the computed optical flow vector field to the predicted heatmap of the surrounding frames. We denote the optical flow transformation as ϕ(·, ·), which outputs optical flow morphed heatmaps of input heatmaps **f**^*k*^ with reference to target frame heatmaps **f**^*t*^: *ϕ*(**f**^*k*^, **f**^*t*^) = **o**^*k*^. Note that **f**^*t*^ = ϕ(**f**^*t*^, **f**^*t*^) = **o**^*t*^. We passed all of the optical flow morphed heatmaps through a 1 × 1 convolution layer to get the final output heatmaps of the entire model: $\text{y}\;=conv_{1x1}\left({\left\{{\text{o}}^{k}\right\}}_{k\;\in \;\left\{t-s\times f\;...\;t\;...\;t+s\times f\right\}}\right)$. This 1 × 1 convolution essentially acted as a weighted sum of all the optical flow morphed heatmaps. Finally, we got the predicted pixel location of the *p*^*th*^ key point, denoted Ŷ_*p*_, by getting the global maximum of the *p*^*th*^ final output heatmap **y**_*p*_, and calculated aPCK calculations can be done by comparing Ŷ_*p*_ against *Y*_*p*_.

### Datasets

We did not collect new behavioural datasets of animals for the current study; all animal data were obtained previously for other studies. Our multi-species datasets cover some commonly used animal body plans in locomotion experiments: mouse (*Mus musculus*), zebrafish (*Danio rerio*), fruit fly (*Drosophila melanogaster*), and monkey (*Macaca mulatta*). The mouse dataset was acquired on a LocoMouse setup (Machado et al., 2015), which contains a straight corridor and a bottom mirror that permits observation of side and bottom-views of the mouse with a single camera. The zebrafish dataset was recorded with a camera mounted above a fish tank to film the activity of multiple fish. The fruit fly dataset was downloaded from the Princeton Neuroscience Institute (http://arks.princeton.edu/ark:/88435/dsp01pz50gz79z) (Pereira et al., 2019), and then converted to a compatible format. The monkey dataset was obtained with a camera filming the facial behaviour of a rhesus monkey, with an installed lick port. The detailed datasets attributes are in Supplementary Table 1 and dataset examples in Supplementary Figure 4.

### Dataset Preprocessing

The raw dataset was first split into train, validation, and test sets, with roughly a 3:1:1 ratio (Supplementary Table 2). Best practise for splitting the dataset should ensure abundance in the train set, as well as diversity and independence amongst all three sets, allowing for optimal generalisation on future data without further training. The diversity and independence are preferably achieved by having multiple individuals in the train set, and novel individuals in the validation and test set.

The validation and test sets were ready after resizing to the target dimension, while the train sets required further processing. Three major steps were performed on the train set of each dataset before being used for the data generators of the base models including: augmentation, resizing image and labels, as well as label conversion.

1. To augment all images and labels within the training set with random rotation and flipping. Images were kept without cropping and padded with black background, thus retaining all image information. For our datasets, the angle range for random rotation was (−10°, 10°). No flipping was applied to the mouse or fruit fly train sets; the monkey train set was randomly flipped about the y-axis, and the zebrafish train set was randomly flipped about all axes (x-axis, y-axis, xy-axis).

2. To resize all augmented images and labels to the same size. Images were resampled using pixel area relation, a preferred method for decimating images (Bradski, 2000). The same transformations were also applied to the labels.

3. To convert human defined labels to heatmaps. The heatmap primarily functions to distinguish points closer to the ground truth, as they have a higher weight than points at greater distance, even if all points in the heatmap are considered “correct.” Normalisation to account for the various scales of the animal and image will happen by default in the human labelling process as users can specify the label size. Human defined labels were either pixel coordinates or bounding boxes. For the heatmap of each key point, a 2D tensor of image size was initialised with all zeros. The heatmap was of corresponding image size, so small key points such as mouse paws did not get shrunk to a single pixel. A 2D Gaussian distribution was generated by probability density function (PDF) within a user defined area or the bounding box area on the tensor, with the centre of the area being the ground truth location for the joint and location of peak value for the 2D Gaussian distribution. Then, the heatmap was normalised to make the maximum value 1.0, and any value on the heatmap smaller than 0.1 was set to 0. The third step was the most crucial step as it is closely tied to the eventual evaluation using aPCK, because the area defined by the labeller for the 2D Gaussian distribution will be the area considered as accepted or “correct.” Note that all bounding box sizes and shapes were chosen based on the features of the key points in the images.

Since the datasets were too large to be directly stored in memory, train and validation sets were converted to data generators before feeding them into the model. For each base model, a single training input consisted of a batch of images converted to tensors and a batch of multiple copies of label heatmap tensor, depending on number of stages. The tensors were multiplied by a user defined peak value to increase contrast between label region and remaining pixels. From our training experience, most models cannot be trained without this process.

To train the OpticalFlow model, we first needed an ordered sequence of 2*n* + 1 images to be used as inputs to a pre-trained base model. The base model produced an ordered sequence of 2*n* + 1 heatmap tensors as outputs, which were then fed into the OpticalFlow model. The labels for the OpticalFlow training process were a single set of heatmap labels in the same format as the labels for base models.

### Base Model Training Setup

To make fair comparisons between various base model designs, we set the training length at 50 epochs and the batch size at 10 for all base models on all datasets. We also tried to keep a constant learning rate of 0.0001 across different models and datasets. However, when using the LEAP model, a learning rate of 0.0001 led to wrong predictions on one of the key points for the mouse bottom-view dataset. We therefore used a learning rate of 0.0003 instead in this specific instance. All heatmap labels had a truncated normal distribution with peak value of 16 located at the manually labelled key point position. All models were trained using an ADAM (Kingma and Ba, 2015) optimizer with beta1 = 0.9, beta2 = 0.999, and no decay. The loss function for all models was mean squared error (MSE), but all results were evaluated using aPCK for reasons mentioned before. An overall MSE value of all frames and key points is automatically reported for each training iteration to help track training progress. We are aware of the fact that DeepLabCut did not originally use MSE as its loss function, but later experiments have shown that DeepLabCut results do not change very much with MSE as loss function (Graving et al., 2019). Thus, for fair comparison of the model structure, with all other factors constant, we decided to use MSE as the loss function for all models. For comparisons between models, we used paired *t*-tests, paired by key points, to confirm the significance of the differences between models.

### Base Model Implementation

To make fair comparisons between base model designs, all training, data generation and evaluation procedures were identical. All base models were implemented using Keras (Chollet, 2015) and are available on Github.

The standard FlexibleBaseline models used in model comparisons all had the same hyperparameter: ImageNet pre-trained ResNet50 backbone outputs after Conv4 block and the filter number for the last 3 transposed convolution layers are 64, 64, and 2× number of key points respectively. There was an intermediate supervision after Conv3 block of ResNet50 backbone.

LEAP models were implemented exactly based on the specification of the original paper. Since LEAP also produces heatmaps of original image size, our data generation process worked perfectly with the model.

DeepLabCut models were also implemented according to the original paper, except the original hyperparameters produced prediction heatmaps were smaller than the original images. For DeepLabCut to train using the same data generation process, we changed the kernel size and stride of the final transpose convolution layer to 36 × 36 and 32 × 32, respectively.

DeepPoseKit was originally implemented using Keras (Graving et al., 2019), so our StackedDenseNet implementation was nearly identical to the DeepPoseKit Github implementation, with some minor refactoring to ensure the model works with the rest of the code base. Since the original Stacked DenseNet also produced prediction heatmaps of a smaller size, an additional TransitionUp module (from DenseNet) was added before each output layer to ensure the model produced outputs of original image size.

### Flexibility Comparison Setup

The 3 versions of FlexibleBaseline had hyperparameters specified in Supplementary Table 3. For fruit fly datasets, all model and dataset combinations were trained with 8,000 images randomly sampled with replacement from their respective training set. For mouse side-view datasets, all model and dataset combinations were trained with 400,000 images randomly sampled with replacement from their respective training set.

### OpticalFlow Implementation and Setup

Our OpticalFlow model was similar in principle to a component of Flowing ConvNet (Pfister et al., 2015), but had major changes in implementation to allow for skip ratio and predefined frame range. The hyperparameter values for OpticalFlow from Farneback algorithm are: window size of 27 pixels, pyramid scale of 0.5 with 5 levels and 8 iteration on each pyramid level; pixel neighbourhood size was 7 for polynomial expansion, with a corresponding *poly_sigma* of 1.5.

In our OpticalFlow models comparisons, all OpticalFlow models were trained for 30 epochs with a skip ratio of 1. All OpticalFlow models had a learning rate of 0.0001, except for StackedDenseNet, which had a learning rate of 0.00015. The OpticalFlow model for StackedDenseNet used a slightly higher learning rate, because its validation curve did not plateau with a learning rate of 0.0001 after 30 epochs. All OpticalFlow models had a frame range of 4, except for LEAP, which had a frame range of 2. The LEAP base model had many more prediction errors than other base models, so including more frames often introduced false information to the target frame. Hyperparameters for the OpticalFlow models are summarised in Supplementary Table 4.

### Computing Environment

All training and inference were done on VM instances from Computing Engine of Google Cloud with identical configuration. Each VM instance was a general-purpose N1 series machine with 24 vCPU, 156GB of memory and 2 Nvidia Tesla V100 16GB VRAM GPU. The OS image on each instance was “Deep Learning Image: TensorFlow 1.13.1 m27” with CUDA 10.0 installed.

## Data Availability Statement

The original contributions presented in the study are publicly available. This data can be found here: The software package for OptiFlex is available at https://github.com/saptera/OptiFlex.

## Ethics Statement

Ethical review and approval was not required for the animal study because all animal data used in this paper were videos, which were provided by other research groups or acquired from previously published data.

## Author Contributions

XL and SY constructed all main computational components. XL, SY, and CD provided the concepts and background for the current work. TH, SL, NF, SY, and MK provided all the video data and annotations. XL, SY, TH, and CD wrote the manuscript. CD provided the financial means of the project and supervised it. All authors contributed to the final version.

## Conflict of Interest

The authors declare that the research was conducted in the absence of any commercial or financial relationships that could be construed as a potential conflict of interest.
