# Supplementary material for: OptiFlex: Multi-Frame Animal Pose Estimation Combining Deep Learning With Optical Flow
Source: Front Cell Neurosci. 2021 May 28;15:621252. doi: 10.3389/fncel.2021.621252 (PMC8194069; doi:10.3389/fncel.2021.621252)
Supplement: Supplementary file 7 [file Data_Sheet_1.pdf]

Supplementary Information

Table S1, Dataset attributes.

Table S2, Training dataset division.

Table S3, Hyperparameters of FlexibleBaseline versions.

Table S4, Hyperparameters for Optical flow models.

Table S5(A), Comparison between BaseModel and OpticalFlow prediction aPCK error rates on [mouse side-view] dataset.

Table S5(B), Paired t-tests p-values of OpticalFlow comparison results on [mouse side-view] dataset based on aPCK.

Table S6(A), Comparison between BaseModel and MultiviewCorrection prediction aPCK error rates on [mouse side-view] dataset.

Table S6(B), Paired t-tests p-values of MultiviewCorrection comparison results on [mouse side-view] dataset based on aPCK.

Tables S7(A)-(E), BaseModel prediction aPCK error rates on different datasets.

Table S7(F), Paired t-tests p-values of FlexibleBaseline vs. models based on aPCK.

Tables S8(A)-(B), Comparison between FlexibleBaseline versions' prediction aPCK error rates on sampled [mouse side-view] and [fruit fly] datasets.

Table S9 and Figure S1, Comparison between fully trained FlexibleBaseline versions' prediction aPCK error rates on [mouse side-view] dataset.

Figure S2, Manual labelling GUI.

Figure S3, Dataset creation GUI.

Figure S4, Dataset examples.

Video S1, Compare prediction results of FlexibleBaseline /w OpticalFlow.

Video S2, Compare prediction results of FlexibleBaseline /w MultiviewCorrection.

Videos S3(A)-(D), Compare prediction results of different base models.

TABLE S1 | Dataset attributes.

Dataset	Image Size (w × h)	Frame Rate (fps)	Total # of Frames	# of Key Points
Mouse (Side)	512 × 128	400 reduced to 100	5134	8
Mouse (Bottom)	512 × 128	400 reduced to 100	5134	8
Fruit Fly	512 × 256	100	1500	32
Monkey	512 × 256	40	391	7
Zebrafish	512 × 256	25	451	12

TABLE S2 | Training dataset division.

Dataset	Train Set Image #	Validation Set Image #	Test Set Image #
Mouse (Side)	2599	1207	1051
Mouse (Bottom)	2606	1325	1203
Fruit Fly	900	300	300
Monkey	235	78	78
Zebrafish	271	90	90

All images in train set were augmented 8 times. The table shows the number of images before augmentation.

TABLE S3 | Hyperparameters of FlexibleBaseline versions.

FlexibleBaseline Version	ResNet50 Backbone Output	Intermediate Supervision	# Filters in 1st TrsConv	# Filters in 2nd TrsConv	# Filters in 3rd TrsConv
Standard	after Conv4	after Conv3	64	64	2x # key points
Reduced	after Conv3	after Conv2	64	64	2x # key points
Small	after Conv2	N/A	32	32	2x # key points

Learning rate for all versions is 0.0001.
Conv stands for “Convolutional Layer”; TrsConv stands for “Transposed Convolutional Layer”.

TABLE S4 | Hyperparameters for OpticalFlow models.

Hyperparameter	FlexibleBaseline	DeepLabCut	LEAP	StackedDenseNet
Skip Ratio	1	1	1	1
Frame Range	4	4	2	4
Learning Rate	0.0001	0.0001	0.0001	0.00015

TABLE S5A | Comparison between BaseModel and OpticalFlow prediction aPCK error rates on [mouse side-view] dataset.

Joint Name	FlexibleBaseline		DeepLabCut		LEAP		StackedDenseNet	
	BaseModel	OpticalFlow	BaseModel	OpticalFlow	BaseModel	OpticalFlow	BaseModel	OpticalFlow
Front Right Paw	0.076118	0.070409	0.086584	0.085633	0.214082	0.206470	0.264510	0.255947
Hind Right Paw	0.111323	0.099905	0.131304	0.121789	0.169363	0.167460	0.210276	0.190295
Front Left Paw	0.126546	0.114177	0.163654	0.151284	0.238820	0.219791	0.198858	0.185538
Hind Left Paw	0.092293	0.085633	0.172217	0.153187	0.171265	0.161751	0.206470	0.186489
Snout	0.093245	0.085633	0.089439	0.084681	0.125595	0.112274	0.103711	0.097050
Tail 01	0.035205	0.031399	0.055186	0.054234	0.127498	0.129401	0.058991	0.056137
Tail 02	0.213130	0.213130	0.265461	0.263559	0.381541	0.362512	0.242626	0.247383
Tail 03	0.083730	0.065652	0.087536	0.070409	0.080875	0.065652	0.099905	0.098953
BEST COUNT	1	**7**	0	1	0	1	0	0
MEAN	0.103949	**0.095742**	0.131423	0.123097	0.188630	0.178164	0.173168	0.164724
SD	**0.051610**	0.053491	0.067803	0.067431	0.092872	0.089666	0.075159	0.073106

*Best results at each joint are marked as GREEN, best statistical results are marked as **GREEN_BOLD**.*

TABLE S5B | Paired t-tests p-values of OpticalFlow comparison results on [mouse side-view] dataset based on aPCK.

	FlexibleBaseline	DeepLabCut	LEAP	StackedDenseNet
**vs. SELF + OpticalFlow**	**0.00439**	**0.01450**	**0.00625**	**0.03141**
**vs. OptiFlex (FlexibleBaseline + OpticalFlow)**	N/A	**0.00628**	**0.00143**	**0.00948**

SELF is the name of the base model of each column.

***ORANGE** values are $p < 0.05$, **GREEN** values are $p < 0.01$.*

For the last row, significant levels have been corrected with Bonferroni corrector of 3 (3 tests against FlexibleBaseline + OpticalFlow).

TABLE S6A | Comparison between BaseModel and MultiviewCorrection prediction aPCK error rates on [mouse side-view] dataset.

**Data Size**	**FlexibleBaseline**			**DeepLabCut**			**LEAP**			**StackedDenseNet**		
**Joint Name**	**BaseM**	**MVC**	**OF-MVC**	**BaseM**	**MVC**	**OF-MVC**	**BaseM**	**MVC**	**OF-MVC**	**BaseM**	**MVC**	**OF-MVC**
Front Right Paw	0.076118	0.067555	0.067555	0.086584	0.086584	0.087536	0.214082	0.190295	0.198858	0.264510	0.231208	0.227402
Hind Right Paw	0.111323	0.101808	0.095147	0.131304	0.128449	0.119886	0.169363	0.153187	0.156993	0.210276	0.190295	0.172217
Front Left Paw	0.126546	0.106565	0.104662	0.163654	0.145576	0.132255	0.238820	0.220742	0.202664	0.198858	0.176023	0.159848
Hind Left Paw	0.092293	0.089439	0.084681	0.172217	0.174120	0.164605	0.171265	0.159848	0.151284	0.206470	0.182683	0.165557
Snout	0.093245	0.093245	0.085633	0.089439	0.089439	0.084681	0.125595	0.125595	0.112274	0.103711	0.103711	0.097050
Tail 01	0.035205	0.035205	0.031399	0.055186	0.055186	0.054234	0.127498	0.127498	0.129401	0.058991	0.058991	0.056137
Tail 02	0.213130	0.213130	0.213130	0.265461	0.265461	0.263559	0.381541	0.381541	0.362512	0.242626	0.242626	0.247383
Tail 03	0.083730	0.083730	0.065652	0.087536	0.087536	0.070409	0.080875	0.080875	0.065652	0.099905	0.099905	0.098953
BEST COUNT	1	2	**7**	0	0	1	0	0	1	0	0	0
MEAN	0.103949	0.098835	**0.093482**	0.131423	0.129044	0.122146	0.188630	0.179948	0.172455	0.173168	0.160680	0.153068
SD	0.051610	**0.051418**	0.053296	0.067803	0.067044	0.067399	0.092872	0.091848	0.088883	0.075159	0.066080	0.065956

BaseM = BaseModel; MVC = MultiviewCorrection; OF-MVC = BaseModel + OpticalFlow + MultiviewCorrection.

*Best results at each joint are marked as GREEN, best statistical results are marked as **GREEN_BOLD**.*

TABLE S6B | Paired t-tests p-values of MultiviewCorrection comparison results on [mouse side-view] dataset based on aPCK.

	**FlexibleBaseline**	**DeepLabCut**	**LEAP**	**StackedDenseNet**
**vs. SELF + MVC**	0.08419	0.33319	**0.04168**	**0.03836**
**vs. SELF + OpticalFlow + MultiviewCorrection**	**0.00559**	**0.04452**	**0.00334**	**0.02614**
**vs. OptiFlex + MultiviewCorrection**	N/A	**0.00534**	**0.00140**	**0.00924**

SELF is the name of the base model of each column; OptiFlex = FlexibleBaseline + OpticalFlow.

ORANGE *values are $p < 0.05$,* ***GREEN*** *values are $p < 0.01$.*

For the last row, significant levels have been corrected with Bonferroni corrector of 3 (3 tests against FlexibleBaseline + OF-MVC).

TABLE S7A | BaseModel prediction aPCK error rates on [mouse side-view] dataset.

Joint Name	FlexibleBaseline	DeepLabCut	LEAP	StackedDenseNet
Front Right Paw	0.076118	0.086584	0.214082	0.264510
Hind Right Paw	0.111323	0.131304	0.169363	0.210276
Front Left Paw	0.126546	0.163654	0.238820	0.198858
Hind Left Paw	0.092293	0.172217	0.171265	0.206470
Snout	0.093245	0.089439	0.125595	0.103711
Tail 01	0.035205	0.055186	0.127498	0.058991
Tail 02	0.213130	0.265461	0.381541	0.242626
Tail 03	0.083730	0.087536	0.080875	0.099905
BEST COUNT	**6**	1	1	0
MEAN	**0.103949**	0.131423	0.188630	0.173168
SD	**0.051610**	0.067803	0.092872	0.075159

*Best results at each joint are marked as GREEN, best statistical results are marked as **GREEN_BOLD**.*

TABLE S7B | BaseModel prediction aPCK error rates on [mouse bottom-view] dataset.

Joint Name	FlexibleBaseline	DeepLabCut	LEAP	StackedDenseNet
Front Right Paw	0.067332	0.084788	0.085619	0.064838
Hind Right Paw	0.018288	0.016625	0.029094	0.019119
Front Left Paw	0.031588	0.048213	0.087282	0.055694
Hind Left Paw	0.024106	0.044057	0.059850	0.064838
Snout	0.266002	0.278470	0.286783	0.325852
Tail 01	0.024106	0.028263	0.050707	0.085619
Tail 02	0.285952	0.289277	0.448878	0.245220
Tail 03	0.172901	0.185370	0.231089	0.152951
BEST COUNT	**4**	1	0	3
MEAN	**0.111284**	0.121883	0.159913	0.126766
SD	0.113625	0.112932	0.148569	**0.107141**

*Best results at each joint are marked as GREEN, best statistical results are marked as **GREEN_BOLD**.*

TABLE S7C | BaseModel prediction aPCK error rates on [fruit fly] dataset.

Joint Name	FlexibleBaseline	DeepLabCut	LEAP	StackedDenseNet
head	0.000000	0.003333	0.000000	0.000000
eyeL	0.000000	0.003333	0.000000	0.000000
eyeR	0.000000	0.003333	0.000000	0.000000
neck	0.000000	0.003333	0.000000	0.003333
thorax	0.000000	0.003333	0.000000	0.003333
abdomen	0.000000	0.000000	0.000000	0.003333
forelegR1	0.006667	0.003333	0.003333	0.006667
forelegR2	0.023333	0.030000	0.033333	0.023333
forelegR3	0.050000	0.043333	0.056667	0.046667
forelegR4	0.043333	0.043333	0.046667	0.046667
midlegR1	0.013333	0.013333	0.016667	0.016667
midlegR2	0.006667	0.013333	0.006667	0.016667
midlegR3	0.013333	0.013333	0.023333	0.013333
midlegR4	0.023333	0.023333	0.056667	0.033333
hindlegR1	0.006667	0.003333	0.006667	0.003333
hindlegR2	0.056667	0.060000	0.066667	0.056667
hindlegR3	0.066667	0.086667	0.090000	0.083333
hindlegR4	0.140000	0.140000	0.190000	0.190000
forelegL1	0.003333	0.003333	0.003333	0.006667
forelegL2	0.033333	0.033333	0.040000	0.033333
forelegL3	0.036667	0.036667	0.046667	0.030000
forelegL4	0.036667	0.040000	0.063333	0.033333
midlegL1	0.010000	0.006667	0.010000	0.006667
midlegL2	0.023333	0.030000	0.036667	0.030000
midlegL3	0.046667	0.033333	0.033333	0.040000
midlegL4	0.033333	0.036667	0.053333	0.033333
hindlegL1	0.010000	0.010000	0.010000	0.013333
hindlegL2	0.060000	0.056667	0.056667	0.076667
hindlegL3	0.050000	0.056667	0.066667	0.040000
hindlegL4	0.090000	0.063333	0.133333	0.110000
wingL	0.000000	0.003333	0.006667	0.000000
wingR	0.000000	0.000000	0.006667	0.000000
BEST COUNT	**22**	17	12	15
MEAN	**0.027604**	0.028125	0.036354	0.031250
SD	0.031557	**0.030702**	0.042429	0.039520

*Best results at each joint are marked as GREEN, best statistical results are marked as **GREEN_BOLD**.*

TABLE S7D | BaseModel prediction aPCK error rates on [monkey] dataset.

Joint Name	FlexibleBaseline	DeepLabCut	LEAP	StackedDenseNet
upperlip1	0.000000	0.000000	0.000000	0.000000
upperlip2	0.000000	0.000000	0.000000	0.000000
lowerlip1	0.000000	0.000000	0.000000	0.000000
lowerlip2	0.012821	0.012821	0.025641	0.012821
brow	0.000000	0.000000	0.000000	0.000000
lickspout	0.000000	0.000000	0.000000	0.000000
tongue	0.012821	0.012821	0.012821	0.038462
BEST COUNT	**7**	**7**	6	6
MEAN	**0.003663**	**0.003663**	0.005495	0.007326
SD	**0.006256**	**0.006256**	0.010087	0.014537

*Best results at each joint are marked as GREEN, best statistical results are marked as **GREEN_BOLD**.*

TABLE S7E | BaseModel prediction aPCK error rates on [zebrafish] dataset.

Joint Name	FlexibleBaseline	DeepLabCut	LEAP	StackedDenseNet
zf_01	0.111111	0.111111	0.133333	0.177778
zf_02	0.077778	0.155556	0.333333	0.733333
zf_03	0.100000	0.111111	0.588889	0.777778
zf_04	0.244444	0.222222	0.611111	0.733333
zf_05	0.033333	0.033333	0.155556	0.055556
zf_06	0.188889	0.211111	0.433333	0.844444
zf_07	0.222222	0.277778	0.622222	0.877778
zf_08	0.088889	0.166667	0.377778	0.700000
zf_09	0.055556	0.044444	0.288889	0.300000
zf_10	0.155556	0.155556	0.344444	0.200000
zf_11	0.055556	0.055556	0.344444	0.166667
zf_12	0.122222	0.300000	0.611111	0.533333
BEST COUNT	**10**	6	0	0
MEAN	**0.121296**	0.153704	0.403704	0.508333
SD	**0.068076**	0.087724	0.173065	0.306023

*Best results at each joint are marked as GREEN, best statistical results are marked as **GREEN_BOLD**.*

TABLE S7F | Paired t-tests p-value of FlexibleBaseline vs. models based on aPCK.

Dataset	vs. DeepLabCut	vs. LEAP	vs. StackedDenseNet
Mouse Side-view	0.02663	**0.00361**	**0.01613**
Mouse Bottom-view	**0.00622**	0.02682	0.27910
Fruit Fly	0.68889	**0.00118**	0.06442
Monkey	1.00000	0.35592	0.35592
Zebrafish	0.07348	**0.00002**	**0.00045**

***ORANGE** values are $p < 0.05 / 3$, **GREEN** values are $p < 0.01 / 3$.*
Significant levels have been corrected with Bonferroni corrector of 3 (each animal with 3 tests).

TABLE S8A | Comparison between FlexibleBaseline versions' prediction aPCK error rates on sampled [mouse side-view] dataset.

**Data Size**		**300**			**600**			**1200**			**2599 (Full)**	
**Joint Name**	**Standard**	**Reduced**	**Small**	**Standard**	**Reduced**	**Small**	**Standard**	**Reduced**	**Small**	**Standard**	**Reduced**	**Small**
Front Right Paw	0.287345	0.282588	0.228354	0.218839	0.260704	0.268316	0.199810	0.221694	0.249286	0.203616	0.215985	0.235966
Hind Right Paw	0.282588	0.280685	0.253092	0.213130	0.204567	0.211227	0.225500	0.229305	0.148430	0.190295	0.191246	0.184586
Front Left Paw	0.299715	0.287345	0.212179	0.220742	0.229305	0.177926	0.236917	0.215033	0.192198	0.215033	0.166508	0.188392
Hind Left Paw	0.258801	0.203616	0.176974	0.201713	0.216936	0.206470	0.222645	0.189343	0.166508	0.260704	0.183635	0.157945
Snout	0.108468	0.123692	0.077069	0.146527	0.113225	0.080875	0.092293	0.101808	0.099905	0.122740	0.117983	0.108468
Tail 01	0.067555	0.062797	0.036156	0.039010	0.037108	0.030447	0.075167	0.027593	0.022835	0.042816	0.038059	0.034253
Tail 02	0.295909	0.285442	0.343482	0.303520	0.271170	0.381541	0.313987	0.316841	0.405328	0.317793	0.287345	0.359657
Tail 03	0.180780	0.156993	0.129401	0.177926	0.169363	0.094196	0.218839	0.132255	0.095147	0.169363	0.115128	0.120837
BEST COUNT	0	1	**7**	2	2	**4**	3	0	**5**	1	3	**4**
MEAN	0.222645	0.210395	**0.182088**	0.190176	0.187797	**0.181375**	0.198145	0.179234	**0.172455**	0.190295	**0.164486**	0.173763
SD	0.091991	**0.087755**	0.099472	**0.075753**	0.079037	0.113161	**0.078404**	0.089304	0.116350	0.083456	**0.075039**	0.096758

Trained for 40,000 steps at a batch size of 10. *For each group, best results at each joint are marked as GREEN, best statistical results are marked as **GREEN_BOLD**.*

TABLE S8B | Comparison between FlexibleBaseline versions' prediction aPCK error rates on sampled [fruit fly] dataset.

Data Size	10			40			70			100		
**Joint Name**	**Standard**	**Reduced**	**Small**	**Standard**	**Reduced**	**Small**	**Standard**	**Reduced**	**Small**	**Standard**	**Reduced**	**Small**
head	0.000000	0.000000	0.000000	0.000000	0.000000	0.000000	0.000000	0.000000	0.000000	0.000000	0.000000	0.000000
eyeL	0.000000	0.000000	0.000000	0.000000	0.000000	0.000000	0.000000	0.000000	0.000000	0.000000	0.000000	0.000000
eyeR	0.000000	0.000000	0.000000	0.000000	0.000000	0.016667	0.000000	0.000000	0.000000	0.000000	0.000000	0.000000
neck	0.000000	0.000000	0.000000	0.000000	0.000000	0.000000	0.000000	0.000000	0.000000	0.000000	0.000000	0.000000
thorax	0.000000	0.000000	0.003333	0.000000	0.000000	0.003333	0.000000	0.000000	0.003333	0.000000	0.000000	0.000000
abdomen	0.006667	0.000000	0.000000	0.000000	0.000000	0.000000	0.000000	0.000000	0.000000	0.000000	0.000000	0.000000
forelegR1	0.006667	0.010000	0.010000	0.006667	0.006667	0.006667	0.010000	0.010000	0.006667	0.006667	0.006667	0.010000
forelegR2	0.120000	0.130000	0.203333	0.110000	0.123333	0.123333	0.083333	0.093333	0.140000	0.060000	0.053333	0.066667
forelegR3	0.296667	0.313333	0.360000	0.160000	0.203333	0.253333	0.120000	0.163333	0.163333	0.076667	0.100000	0.093333
forelegR4	0.370000	0.256667	0.233333	0.070000	0.110000	0.096667	0.070000	0.106667	0.083333	0.056667	0.080000	0.083333
midlegR1	0.030000	0.043333	0.040000	0.016667	0.023333	0.020000	0.016667	0.030000	0.020000	0.020000	0.020000	0.026667
midlegR2	0.186667	0.083333	0.136667	0.066667	0.020000	0.053333	0.036667	0.030000	0.020000	0.036667	0.036667	0.036667
midlegR3	0.363333	0.370000	0.443333	0.153333	0.096667	0.130000	0.086667	0.053333	0.033333	0.040000	0.043333	0.036667
midlegR4	0.386667	0.243333	0.276667	0.100000	0.110000	0.123333	0.076667	0.106667	0.083333	0.070000	0.063333	0.080000
hindlegR1	0.010000	0.010000	0.023333	0.010000	0.016667	0.013333	0.006667	0.003333	0.013333	0.006667	0.003333	0.020000
hindlegR2	0.260000	0.340000	0.380000	0.123333	0.113333	0.133333	0.110000	0.123333	0.090000	0.070000	0.083333	0.096667
hindlegR3	0.430000	0.276667	0.316667	0.146667	0.270000	0.190000	0.126667	0.156667	0.240000	0.113333	0.186667	0.156667
hindlegR4	0.613333	0.676667	0.633333	0.263333	0.293333	0.286667	0.313333	0.306667	0.366667	0.300000	0.263333	0.280000
forelegL1	0.003333	0.006667	0.010000	0.003333	0.003333	0.003333	0.003333	0.003333	0.006667	0.003333	0.003333	0.010000
forelegL2	0.150000	0.133333	0.206667	0.126667	0.086667	0.156667	0.136667	0.063333	0.070000	0.050000	0.063333	0.080000
forelegL3	0.233333	0.246667	0.233333	0.120000	0.166667	0.156667	0.086667	0.116667	0.143333	0.076667	0.066667	0.096667
forelegL4	0.253333	0.153333	0.213333	0.063333	0.116667	0.140000	0.080000	0.063333	0.113333	0.060000	0.056667	0.053333
midlegL1	0.016667	0.020000	0.020000	0.013333	0.010000	0.013333	0.006667	0.010000	0.016667	0.010000	0.010000	0.010000
midlegL2	0.150000	0.080000	0.120000	0.053333	0.070000	0.083333	0.043333	0.046667	0.100000	0.043333	0.056667	0.053333
midlegL3	0.406667	0.413333	0.420000	0.110000	0.126667	0.116667	0.090000	0.090000	0.093333	0.066667	0.070000	0.086667
midlegL4	0.170000	0.220000	0.180000	0.120000	0.166667	0.126667	0.076667	0.110000	0.106667	0.070000	0.066667	0.083333
hindlegL1	0.010000	0.010000	0.010000	0.010000	0.023333	0.013333	0.010000	0.010000	0.030000	0.010000	0.010000	0.013333
hindlegL2	0.210000	0.330000	0.193333	0.133333	0.200000	0.216667	0.100000	0.126667	0.136667	0.086667	0.093333	0.120000
hindlegL3	0.476667	0.446667	0.226667	0.160000	0.170000	0.213333	0.123333	0.173333	0.220000	0.090000	0.123333	0.116667
hindlegL4	0.460000	0.446667	0.413333	0.186667	0.216667	0.283333	0.206667	0.196667	0.226667	0.163333	0.146667	0.170000
wingL	0.020000	0.026667	0.013333	0.020000	0.010000	0.053333	0.010000	0.016667	0.013333	0.020000	0.043333	0.013333
wingR	0.023333	0.036667	0.026667	0.006667	0.010000	0.016667	0.003333	0.013333	0.006667	0.003333	0.023333	0.013333
BEST COUNT	**20**	14	12	**26**	14	6	**23**	14	9	**22**	19	11
MEAN	0.176979	**0.166354**	0.167083	**0.073542**	0.086354	0.095104	**0.063542**	0.069479	0.079583	**0.050313**	0.055417	0.059583
SD	0.181863	0.178601	**0.170276**	**0.071105**	0.087778	0.090292	**0.070746**	0.074416	0.089454	0.060608	**0.060130**	0.062892

*Trained for 8,000 steps at a batch size of 10. For each group, best results at each joint are marked as GREEN, best statistical results are marked as **GREEN_BOLD**.*

TABLE S9 | Comparison between fully trained FlexibleBaseline versions' prediction aPCK error rates on [mouse side-view] dataset.

Joint Name	Standard	Reduced	Small
Front Right Paw	0.076118	0.117031	0.154139
Hind Right Paw	0.111323	0.146527	0.164605
Front Left Paw	0.126546	0.159848	0.156993
Hind Left Paw	0.092293	0.148430	0.128449
Snout	0.093245	0.085633	0.078021
Tail 01	0.035205	0.055186	0.030447
Tail 02	0.213130	0.256898	0.320647
Tail 03	0.083730	0.121789	0.096099
BEST COUNT	6	0	2
MEAN	**0.103949**	0.136418	0.141175
SD	**0.051610**	0.059855	0.085874

Best results at each joint are marked as GREEN, best statistical results are marked as ***GREEN_BOLD****.*

Training was performed on the full [mouse side-view] dataset (2599 frames), and the training was done with 50 epochs at learning rate of 0.0001, batch size of 10.

FIGURE S1 | Comparison between fully trained FlexibleBaseline versions' prediction aPCK error rate on [mouse side-view] dataset.

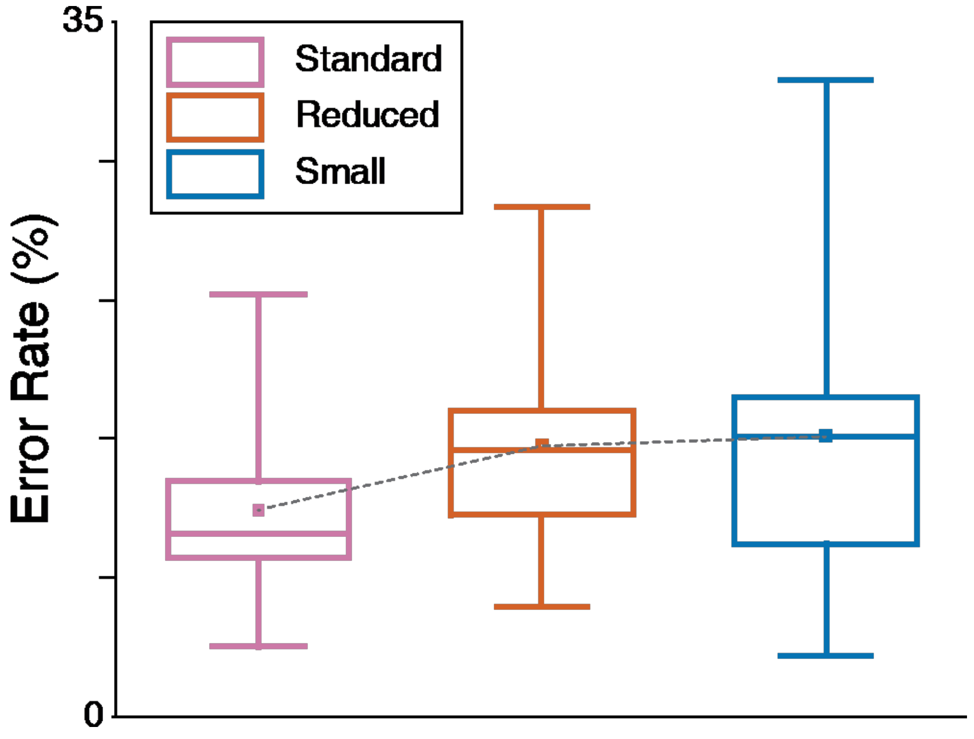

Prediction error rates summarized in box plots with the five number summary being (minimum, first quartile, median, third quartile, maximum). The small square in the middle of each box plot represents the mean error rate.

FIGURE S2 | Manual labelling GUI.

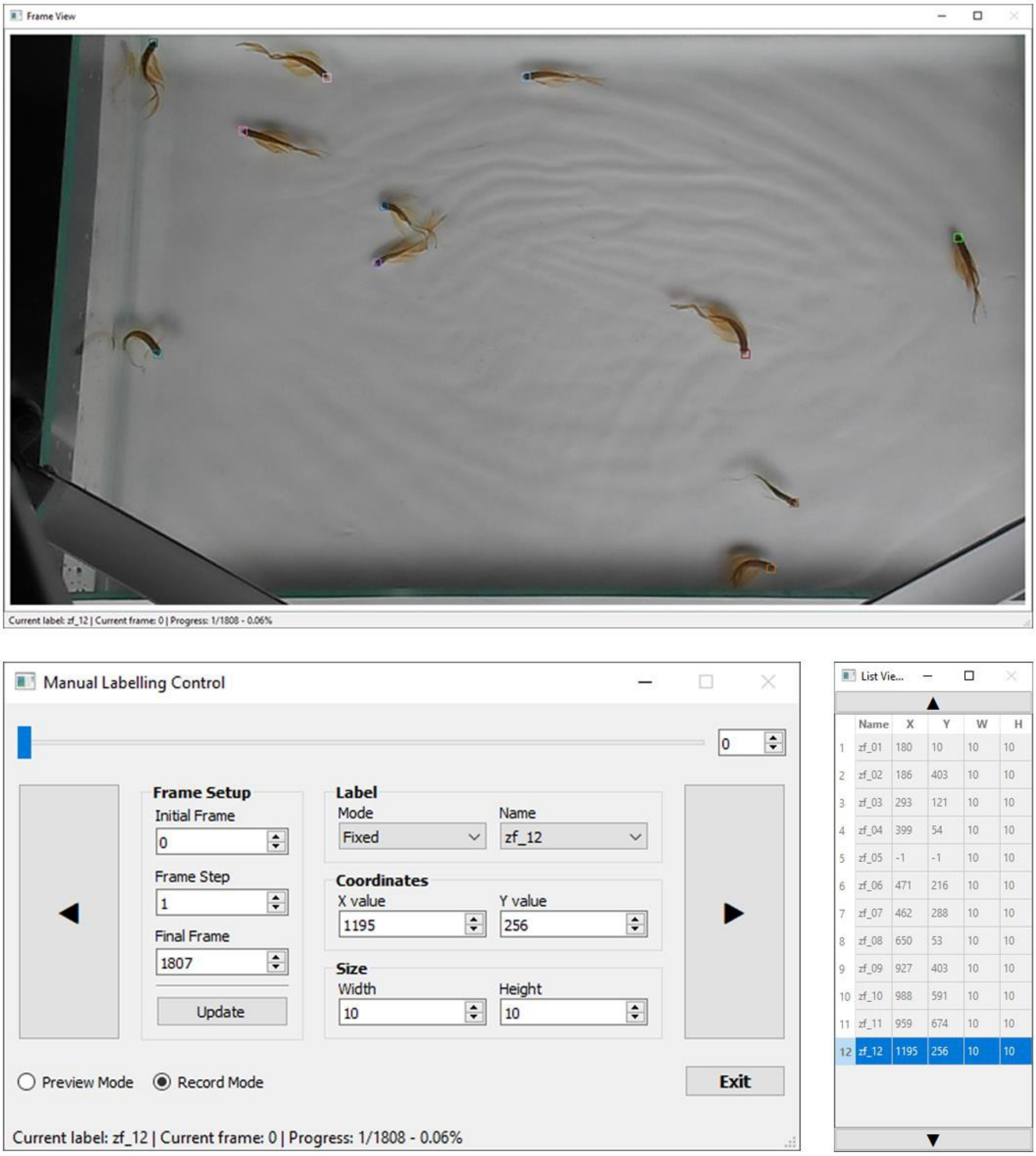

A GUI for manual labelling is included in the code package. Users could use the “A” or “D” key to navigate to the previous or the next frame, the “W” or “S” key to select between labels. Users are also able to select a label with “Left Mouse Click”, add a label with “SHIFT” + “Left Mouse Click”, delete a label with “Right Mouse Click”. Moving a selected label can be achieved with simple “Mouse Dragging”. The windows in this example screenshots have been resized to fit the pages.

FIGURE S3 | Dataset creation GUI.

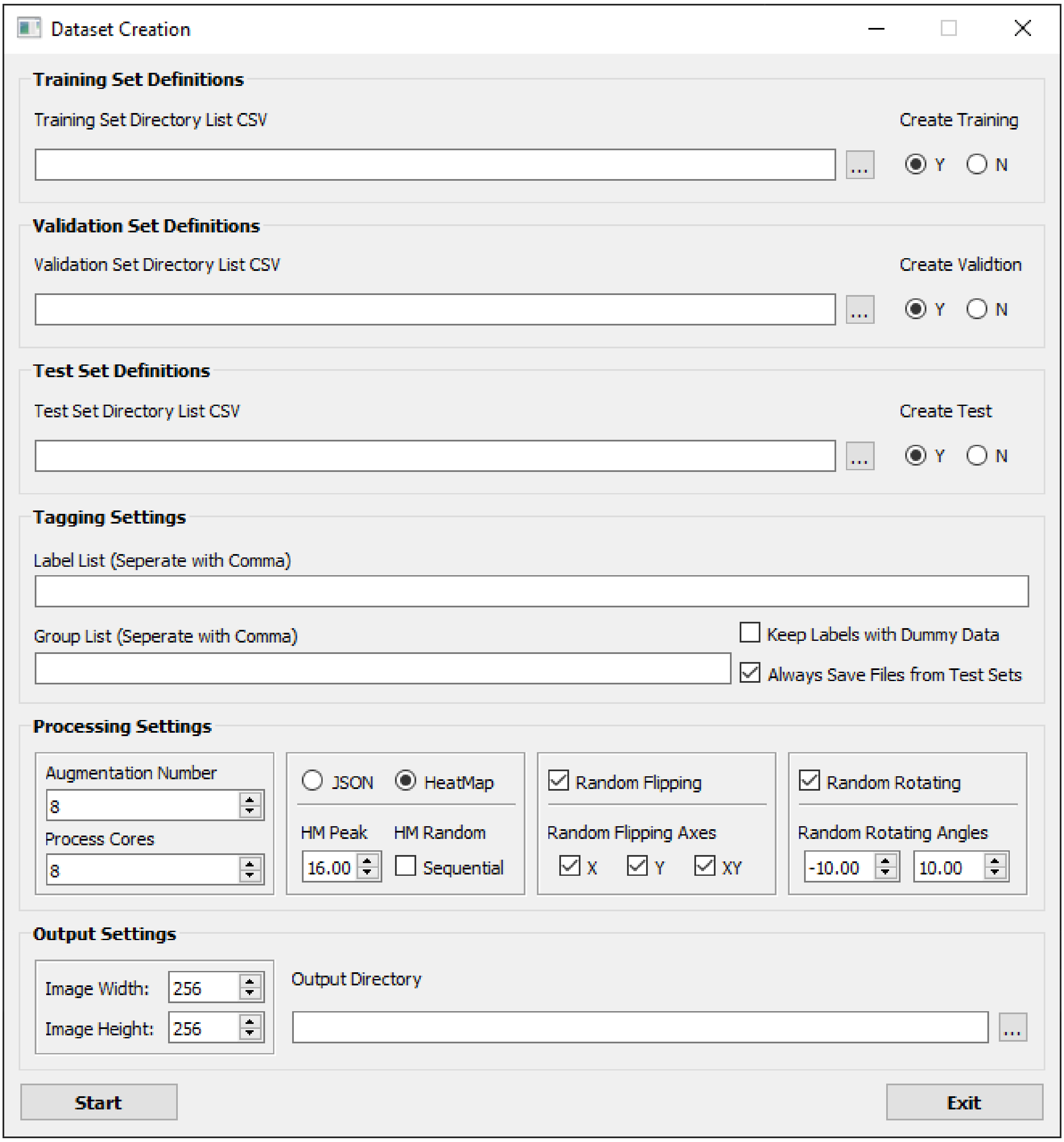

A GUI for creating datasets is included in the code package. This GUI could create training/validation/testing datasets from manual labelled data with many different options to meet different training requirements. All instructions have been clearly noted on the GUI.

FIGURE S4 | Dataset examples.

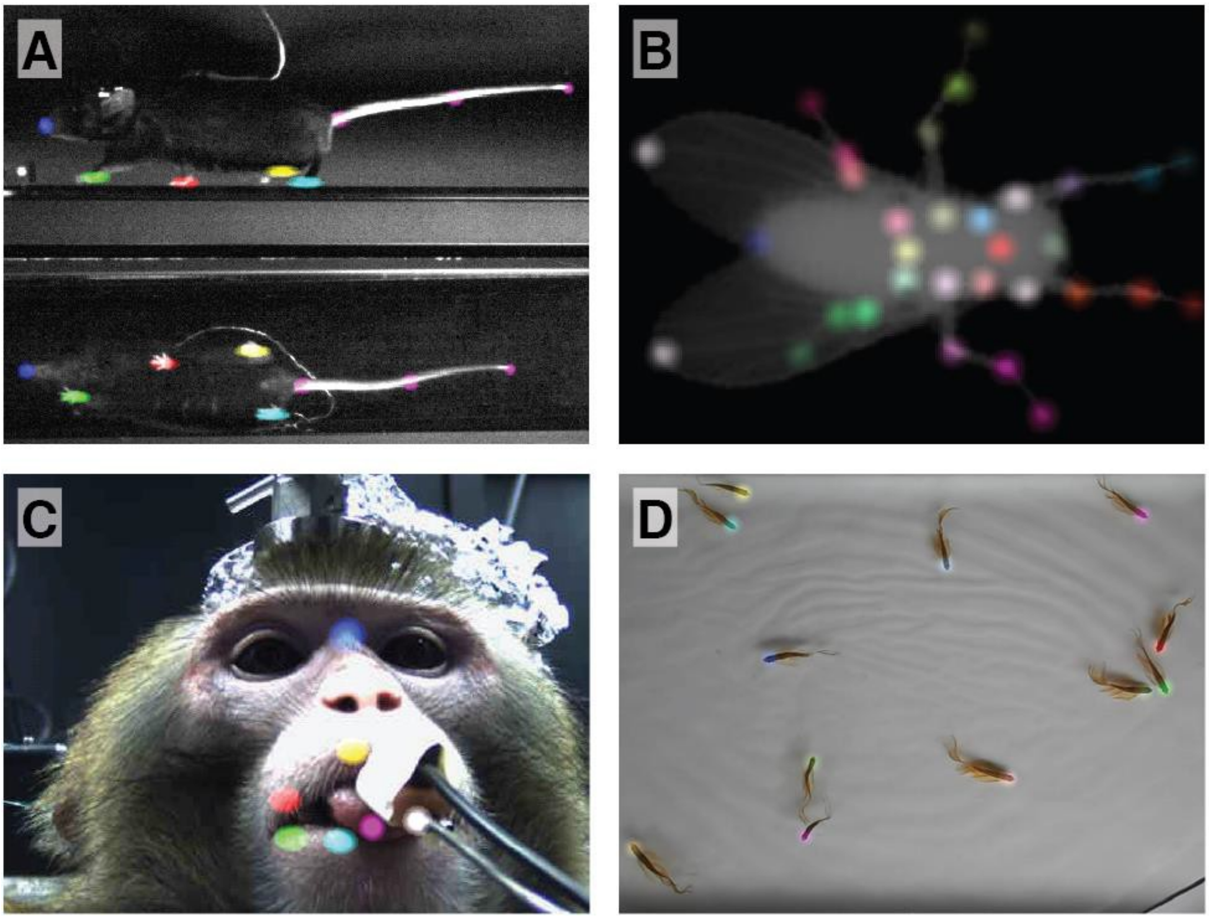

(A), Sample images from mouse datasets. The upper part of the image is from the mouse side-view dataset and the lower part of the image is from the mouse bottom-view dataset. This image is cropped to make the key points labels more visible. (**B),** A sample image from the fruit fly dataset. This dataset is adopted from LEAP with original labels specified in pixel locations. We converted the pixel locations into heatmaps of sizes based on key points features. (**C),** Sample image from the monkey dataset. This image shows tongue extrusion, although the tongue is not visible in most situations. (**D),** Sample image from the zebrafish dataset. All 12 zebrafish are visible in this image, but sometimes they can be hidden from the camera. This image is cropped to make the key points labels more visible.

Note for visualization scheme of videos | For all the videos below, model predictions, evaluated with aPCK, are marked on frames as boxes of 2×2 pixels with line thickness of 2. The boxes merely visualize the outputs of each model; they do not indicate the exact size and shape of the original labels. Boxes corresponding to correct predictions, according to aPCK, are colored as WHITE and incorrect predictions as **RED**.

VIDEO S1 | Compare prediction results of FlexibleBaseline /w OpticalFlow.

This video compared the predictions of FlexibleBaseline with that of FlexibleBaseline + OpticalFlow. The predictions are presented in 3 formats: heatmap, key point location, and aPCK correctness. The colors in key point location results represent the type of the key points, and the visualization scheme for aPCK results is addressed above.

Filename: Video 1.mp4

VIDEO S2 | Compare prediction results of FlexibleBaseline /w MultiviewCorrection.

This video compared the predictions of FlexibleBaseline, FlexibleBaseline + MultiviewCorrection, and FlexibleBaseline + OpticalFlow + MultiviewCorrection. The visualization scheme for aPCK results is addressed above.

Filename: Video 2.mp4

VIDEOS S3(A)-(D) | Compare prediction results of different base models.

These videos compared the prediction results of FlexibleBaseline with other three models (DeepLabCut, LEAP, and StackedDenseNet) on five different datasets (mouse side-view, mouse bottom-view, fruit fly, monkey, and zebrafish). The visualization scheme for aPCK results is addressed above.

VIDEO S1A | Compare prediction results of mouse dataset. *Filename: Video 3.mp4*

VIDEO S1B | Compare prediction results of fruit fly dataset. *Filename: Video 4.mp4*

VIDEO S1C | Compare prediction results of monkey dataset. *Filename: Video 5.mp4*

VIDEO S1D | Compare prediction results of zebrafish dataset. *Filename: Video 6.mp4*
